# COVID-19 Vaccine Hesitancy Among Chinese Population: A Large-Scale National Study

**DOI:** 10.3389/fimmu.2021.781161

**Published:** 2021-11-29

**Authors:** Jian Wu, Quanman Li, Clifford Silver Tarimo, Meiyun Wang, Jianqin Gu, Wei Wei, Mingze Ma, Lipei Zhao, Zihan Mu, Yudong Miao

**Affiliations:** ^1^ Department of Health Management of Public Health, Zhengzhou University, Zhengzhou, China; ^2^ Henan Provincial People’s Hospital, People’s Hospital of Zhengzhou University, Zhengzhou, China; ^3^ School of Medicine, Southern University of Science and Technology, Shenzhen, China

**Keywords:** COVID-19 vaccine hesitancy, China, primary vaccination, booster vaccination, factors (individual factors, contextual factors)

## Abstract

Globally, vaccine hesitancy is a growing public health problem. It is detrimental to the consolidation of immunization program achievements and elimination of vaccine-targeted diseases. The objective of this study was to estimate the prevalence of COVID-19 vaccine hesitancy in China and explore its contributing factors. A national cross-sectional online survey among Chinese adults (≥18 years old) was conducted between August 6, 2021 and August 9 *via* a market research company. We collected sociodemographic information; lifestyle behavior; quality of life; the knowledge, awareness, and behavior of COVID-19; the knowledge, awareness, and behavior of COVID-19 vaccine; willingness of COVID-19 vaccination; accessibility of COVID-19 vaccination services; skepticism about COVID-19 and COVID-19 vaccine; doctor and vaccine developer scale; and so on. Odds ratios (OR) with 95% confidence intervals (CI) were used to estimate the associations by using logistic regression models. A total of 29,925 residents (48.64% men) were enrolled in our study with mean age of 30.99 years. We found an overall prevalence of COVID-19 vaccine hesitancy at 8.40% (95% CI, 8.09–8.72) in primary vaccination and 8.39% (95% CI, 8.07–8.70) in booster vaccination. In addition, after adjusting for potential confounders, we found that women, higher educational level, married residents, higher score of health condition, never smoked, increased washing hands, increased wearing mask, increased social distance, lower level of vaccine conspiracy beliefs, disease risks outweigh vaccine risk, higher level of convenient vaccination, and higher level of trust in doctor and developer were more willing to vaccinate than all others (all *p* < 0.05). Age, sex, educational level, marital status, chronic disease condition, smoking, healthy behaviors, the curability of COVID-19, the channel of accessing information of COVID-19 vaccine, endorsement of vaccine conspiracy beliefs, weigh risks of vaccination against risks of the disease, making a positive influence on the health of others around you, and lower trust in healthcare system may affect the variation of willingness to take a COVID-19 vaccine (all *p* < 0.05). The prevalence of COVID-19 vaccine hesitancy was modest in China, even with the slight resulting cascade of changing vaccination rates between the primary and booster vaccination. Urgent action to address vaccine hesitancy is needed in building trust in medical personnel and vaccine producers, promoting the convenience of vaccination services, and spreading reliable information of COVID-19 vaccination *via* the Internet and other media.

## Introduction

Vaccination is the most cost-efficient method of avoiding infectious diseases and has been one of the most effective public health interventions to date (1–4). The effectiveness of the COVID-19 vaccination depends solely on its uptake. If there are individuals who are hesitant or unwilling to be immunized, the vaccination coverage will be limited. A study indicated that a refusal rate of more than 10% is estimated to be sufficient to weaken the population benefits of vaccination against COVID-19 (5). Since the global outbreak of COVID-19, researchers from all around the world have been working tirelessly and collaboratively to develop the vaccines against the virus. Numerous types of vaccines are currently available including inactivated vaccines, recombinant protein vaccines, adenovirus vector vaccines, attenuated influenza virus vector vaccines, and nucleic acid (mRNA and DNA vaccines) vaccines (6). However, this global effort might be hampered by vaccine hesitancy despite its availability (7).

Vaccine hesitancy has been identified as one of the greatest threats to public health at a global level (8) and a common phenomenon in the developed world for decades (9–11). However, the prevalence of COVID-19 vaccine hesitancy is chaotic globally, posing a significant obstacle to the global efforts to containing the current COVID-19 pandemic. A recent review of vaccine acceptance rates demonstrated that developed countries such as the USA, France, and Italy generally have expected vaccine acceptance rates of less than 60% (ranging from 53.7% to 58.9%). Meanwhile, low rates of COVID-19 vaccine acceptance were reported in the Middle East, Russia, Africa, and several European countries as well (11). However, the current analytics show that countries like China, Malaysia, and Ecuador are expected to have uptake rates of more than 90% (ranging from 91.3% to 97.0%) (12). In addition, another recent survey in the UK showed that 16.9% of respondents were hesitant about the vaccine (13). While a survey conducted in Hong Kong, China, showed a shift in the predicted uptake rate from 44.2% to 34.8% at different waves of local epidemic among the population (14).

The reasons for COVID-19 vaccination hesitancy are varied and, to some extent, unclear. Earlier research examined the complex nature of vaccine hesitancy by examining the epidemiologic triad of environmental, agent, and host factors (i.e., EAH framework) (15). Environmental factors include public health policies, social factors, and media messaging (16, 17) while the agent (vaccine and disease) factors include the perception of vaccine safety and effectiveness, besides the perceived susceptibility to the disease (18). Host factors are dependent on knowledge, previous experience, and educational and income levels (19). Recent research indicates that vaccine hesitation is frequently framed in terms of complacency, confidence, and convenience (3Cs framework). Vaccine hesitation occurs when there is a low perception of the necessity for vaccination (referred to as complacency), worries about the efficacy and safety of the vaccine (referred to as low confidence), and a lack of vaccine accessibility (referred to as convenience) (20). Based on the frameworks, youth, female gender, low income, low education, high informational reliance on social media, low informational reliance on print and broadcast media, membership of other than white ethnic groups, low perceived risk from COVID-19, and low trust in scientists, medics, and biomedical science, as well as (to a much lesser extent) low trust in government were all recognized as relevant factors that may affect COVID-19 vaccine hesitancy. Similar associations have been observed in countries including Canada, USA, Europe, Australia, Japan, Middle East, Russia, and Africa. The incidence of COVID-19 vaccination hesitancy has been researched, as well as how it is influenced by socioeconomic position, particularly individual psychological characteristics. However, such information is lacking in China.

China may be a world leader in COVID-19 vaccination coverage not only because it constitutes one-fifth of the world’s population but also because the country is becoming increasingly interconnected with the rest of the world. Although more than 62.4% (880 million) of the Chinese population have been officially confirmed to be vaccinated so far, some residents are still hesitant to get vaccinated (21). We therefore sought to expand on the EAH and 3C frameworks in order to better explain COVID-19 vaccine hesitancy among Chinese. Therefore, we conducted a nation-wide survey in 31 provinces across mainland China during the period of primary and booster vaccination of COVID-19 vaccines. We calculated the level of COVID-19 vaccine hesitancy (delay or refusal) in a large sample by analyzing expressed readiness to get an approved vaccination and identifying subgroups within the population and regions where it may be higher. Our major objective was to gain a better understanding of vaccine hesitancy on an individual psychological level to guide methods for increasing vaccination acceptance rates.

## Methods

### Participants and Procedures

**Figure 1:**
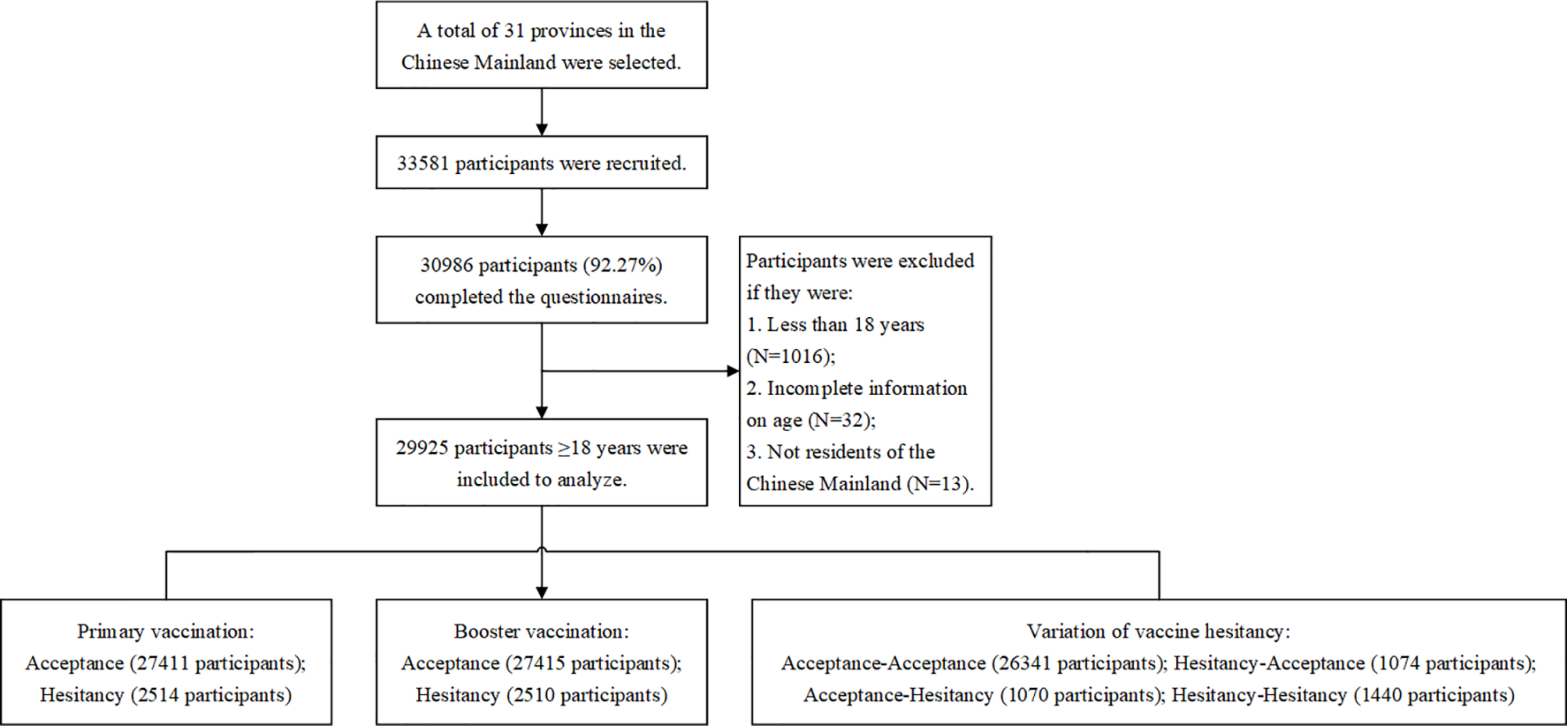
The flowchart of participant selection.

On July 10, 2021, we performed a preliminary online survey in Zhongmou County, Henan Province. We conducted a face-to-face interview with participants from a representative village and community obtained through cluster sampling approach. Basing on the vaccine hesitation rate and the reliability and validity of the questionnaire of the preliminary online survey, we estimated the minimum sample size required for the formal survey to be 6,638 participants, which was based on a prevalence of COVID-19 vaccine hesitancy of 16.57% in a preliminary online survey, an allowable error of 1%, a 95% confidence level, and an anticipated design effect of two. A subsequent national cross-sectional online survey using a snowball sampling method among Chinese adults (≥18 years old) was conducted from August 6, 2021 *via* a market research company. The invited respondents were unaware of the topic prior to their tentative consent to complete the survey. In order to ensure that the sample size for this study was large enough to estimate the prevalence of vaccine hesitancy, a sample saturation was monitored during the investigation. Saturation, in this study, refers to the point at which the sample size reaches a specific size whereby the vaccine hesitancy rate becomes constant and no longer varies considerably with the snowballing sample size growth. We found that when the number of valid questionnaires reached 21,780, the sample began to saturate (**Appendix Figure 1**). We ended the online survey when the valid sample reached 29,925 on August 9, 2021. The flowchart of participant selection and sample saturation monitoring procedures are shown in **Figure 1**.

### Assessments

Due to the absence of a uniform COVID-19 vaccine hesitancy scale in China, we designed two items to assess whether there was a delay in immunization acceptance or refusal based on the Oxford COVID-19 Vaccine Hesitancy Scale (13). The items comprised themes “In terms of COVID-19 vaccination in current stage, I would describe myself as” for estimating hesitancy rate in primary vaccination and “I would describe my attitude towards regularly receiving a COVID-19 vaccine in the future as” for predicting hesitancy rate in booster vaccination in future. For each item, item-specific response options coded from 1 to 5 were used, including (1) Vaccination, (2) Willing to get the COVID-19 vaccine, (3) Delay to getting the COVID-19 vaccine, (4) Unwilling to get the COVID-19 vaccine, and (5) Anti-vaccination.” Higher scores indicated a higher level of vaccine hesitancy. According to the definition of vaccine hesitancy, options (1) and (2) were merged into “Acceptance” and options (3), (4), and (5) were merged into “Hesitancy” during data analysis. Based on the EAH and 3C frameworks, our questionnaire subsequently collected exploratory and confirmatory factors from four aspects, namely, (1) individual characteristics (i.e., social-demographic information, subjective social status, health status), (2) COVID-19 pandemic progress perception (i.e., awareness of COVID-19 blocking, judgement of the trend, pandemic skepticism), (3) COVID-19 vaccine perception (i.e., general knowledge on vaccine, COVID-19 vaccination perception, vaccine skepticism), and (4) the healthcare system dimension (trust in doctors and vaccine developers, convenience of vaccination). All questionnaires are shown in **Appendix 1**.

### Statistical Analysis

The Chi-square goodness-of-fit test was used to monitor sample saturation throughout the formal online survey to determine sample underrepresentation error. An independent samples *t*-test or Chi-square test was carried out to test differences in willingness to get vaccinated across groups. Binary logistic regression analyses were conducted to examine factors associated with COVID-19 vaccine hesitancy and COVID-19 pandemic progress perception, COVID-19 vaccine perception, and trust in healthcare system after controlling for demographic and socioeconomic confounders. Multinomial logistic regression model was applied to assess between associated factors and transformations of COVID-19 vaccine hesitancy. The collinearity test was carried out to assess the correlation between independent variables using a variance inflation factor (VIF) <4, and no collinearity was detected. A sensitivity analysis was performed by excluding participants suffered from chronic diseases to test the robustness of model results and assess source of model uncertainty. We did all statistical analyses using SAS 9.4. Differences were regarded as statistically significant if *p* values were less than 0.05.

### Ethical Approval

This study was deemed exempt by the Life Science Ethics Review Committee of Zhengzhou University.

## Results

### Prevalence of COVID-19 Vaccine Hesitancy

**Figure 2:**
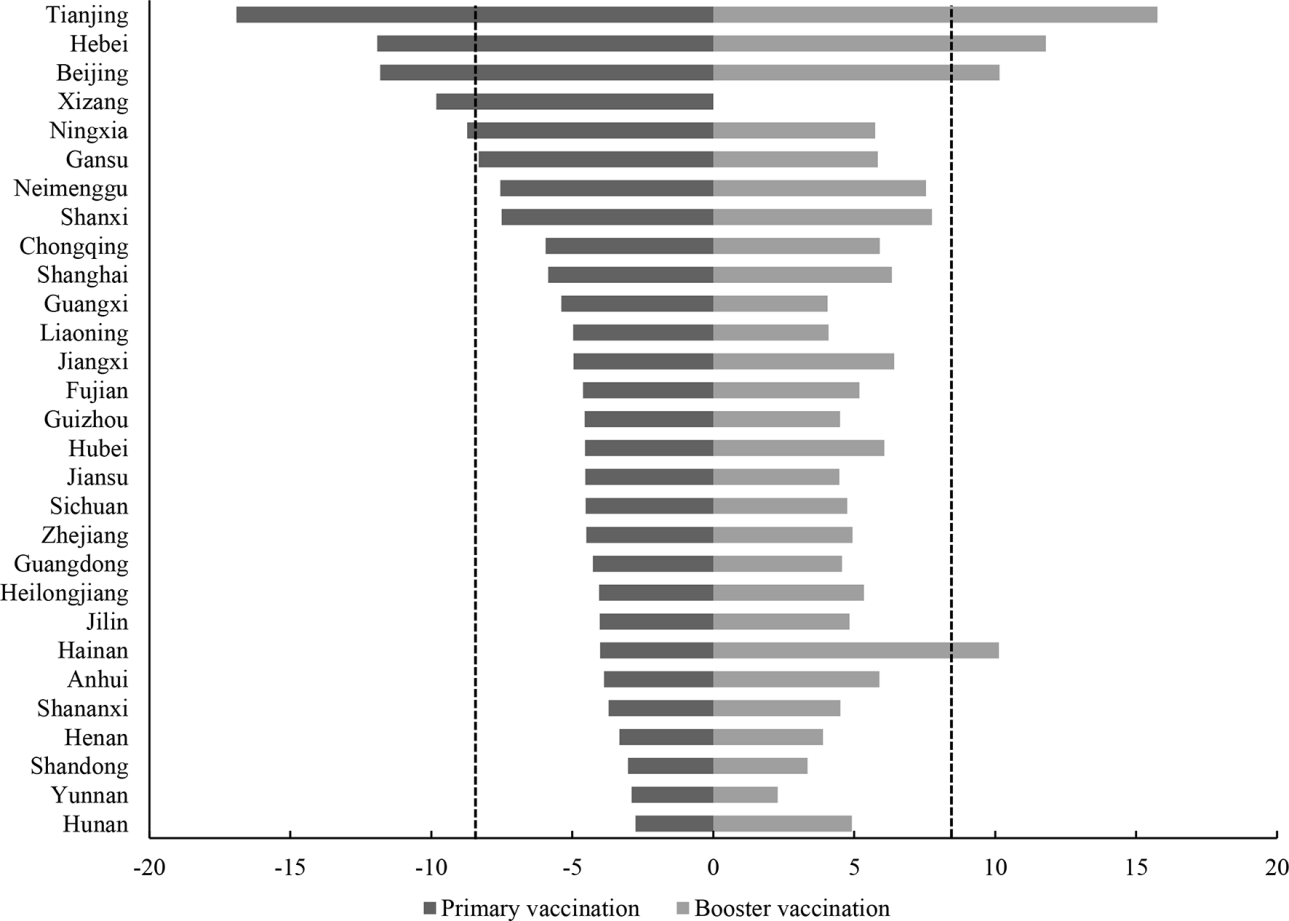
Age- and sex-standardized prevalence of COVID-19 vaccine hesitancy by province in primary vaccination (left) and booster vaccination (right). Qinghai and Xinjiang province were not shown.

A total of 29,925 residents from 31 provinces of Chinese mainland were included in the current study. A summary of the sociodemographic, awareness of COVID-19 pandemic, COVID-19 vaccine exception, trust in healthcare system, and hesitancy of all study participants is provided in **Table 1**. In all, 2,514 (8.40%, 95% confidence interval (CI) 8.09% to 8.72%) participants endorsed clear vaccine hesitancy response in primary vaccination. Furthermore, 2,510 (8.39%, 95% CI 8.07% to 8.70%) expressed their hesitancy in booster vaccination of COVID-19 vaccine. We found that the prevalence of vaccine hesitancy was higher in men than women in all age groups (**Appendix Figure 2**). Higher prevalence in both phrases were observed among population with elders (age ≥60 years), men, lower educational level, minority, religious beliefs, not in marriage, higher subjective social status, lower self-report health condition, suffered from chronic diseases, current smoker, former drinker, extreme endorsement of COVID-19, vaccine conspiracy beliefs, medium or low possibility of curability of COVID-19, inconvenience of vaccination, and lower trust in healthcare system. The hesitancies in both phases varied substantially by the province in mainland China. More than one in 10 of the study population in Beijing, Hebei, and Tianjin were observed to be hesitant to uptake the vaccine in current stage after standardizing age and sex. In terms of predicted hesitancy in the booster vaccination, the age- and sex-standardized prevalence of hesitancy in Beijing, Tianjin, Hebei, and Hainan were more than 10%, ranging from 10.13% to 15.76% (**Figure 2**; **Appendix Table 1**).

**Table 1:** Sociodemographic, awareness of COVID-19 pandemic, COVID-19 vaccine exception, trust in healthcare system, and COVID-19 vaccine hesitancy of all study participants.

Covariates	Total (%)	*p-*value^a^	Vaccine hesitancy in the primary vaccination (95% CI)	*p-*value^a^	Vaccine hesitancy in booster vaccination (95% CI)	*p-*value^a^
Total participants	29,925 (100)		8.40 (8.09–8.72)^b^		8.39 (8.07–8.70)^b^	
**Demographic characteristics**						
Age (years)		<0.001		<0.001		<0.001
18–29	13,312 (44.48)		10.84 (10.31–11.37)^b^		10.97 (10.44–11.50)^b^	
30–39	11,911 (39.80)		6.73 (6.28–7.18)^b^		6.63 (6.19–7.08)^b^	
40–49	3,269 (10.92)		4.68 (3.96–5.40)^b^		4.37 (3.67–5.08)^b^	
50–59	1,149 (3.84)		7.05 (5.57–8.53)^b^		7.57 (6.04–9.10)^b^	
60–	284 (0.95)		12.32 (8.50–16.15)^b^		10.56 (6.99–14.14)^b^	
Sex		<0.001		<0.001		<0.001
Men	14,556 (48.64)		11.55 (8.50–16.15)^b^		11.42 (10.91–11.94)^b^	
Women	15,369 (51.36)		5.42 (5.06–5.78)^b^		5.51 (5.15–5.87)^b^	
Educational status		<0.001		<0.001		<0.001
Below high school	3,839 (12.83)		21.36 (20.06–22.66)^b^		20.08 (18.82–21.35)^b^	
High school graduate	7,893 (26.38)		8.40 (7.79–9.01)^b^		7.99(7.40-8.59)^b^	
University graduate	18,193 (60.80)		5.67 (5.33–6.00)^b^		6.09 (5.74–6.44)^b^	
Ethnic groups		<0.001		<0.001		<0.001
Han	28,579 (95.50)		7.76 (7.45–8.07)^b^		7.86 (7.55–8.17)^b^	
Minority	1,346 (4.50)		21.92 (19.71–24.13)^b^		19.54 (17.42–21.66)^b^	
Religion		<0.001		<0.001		<0.001
Atheist	25,424 (84.96)		6.44 (6.14–6.74)^b^		6.69 (6.39–7.00)^b^	
Others	4,501 (15.04)		19.46 (18.31–20.62)^b^		17.95 (16.83–19.07)^b^	
Marital status		<0.001		<0.001		<0.001
Married	18,363 (61.36)		6.42 (6.06–6.77)^b^		6.33 (5.98–6.69)^b^	
Others	11,562 (38.64)		11.56 (10.97–12.14)^b^		11.65 (11.07–12.24)^b^	
Subjective social status						
Society level	6.66 ± 2.09	<0.001	6.98 ± 2.06^c^	<0.001	6.89 ± 2.02^c^	<0.001
Community level	7.00 ± 2.13	<0.001	7.39 ± 2.14^c^	<0.001	7.27 ± 2.12^c^	<0.001
Self-report health condition (EQ-5D)	84.36 ± 14.58	<0.001	75.55 ± 19.68^c^	<0.001	75.70 ± 19.55^c^	<0.001
Chronic condition		<0.001		<0.001		<0.001
0	24,960 (84.95)		5.48 (5.20–5.77)^b^		5.76 (5.47–6.05)^b^	
1	3,245 (11.04)		22.53 (21.09–23.96)^b^		21.23 (19.83–22.64)^b^	
2	891 (3.03)		22.45 (19.71–25.19)^b^		22.11 (19.39–24.83)^b^	
≥3	287 (0.98)		23.34 (18.45–28.24)^b^		23.34 (18.45–28.24)^b^	
Smoking status		<0.001		<0.001		<0.001
Current smoker	9,702 (32.42)		17.99 (17.22–18.75)^b^		17.13 (16.38–17.88)^b^	
Former smoker	1,664 (5.56)		10.88 (9.38–12.37)^b^		9.19 (7.81–10.58)^b^	
Never smoker	18,559 (62.02)		3.17 (2.92–3.42)^b^		3.74 (3.47–4.02)^b^	
Drinking status		<0.001		<0.001		<0.001
Current drinker	18,484 (61.77)		11.04 (10.58–11.49)^b^		10.91 (10.46–11.36)^b^	
Former drinker	1,080 (3.61)		12.96 (10.96–14.97)^b^		11.39 (9.49–13.28)^b^	
Never drinker	10,361 (34.62)		3.22 (2.88–3.56)^b^		3.57 (3.21–3.93)^b^	
Health behaviors						
Washing hands	23,737 (79.32)	<0.001	4.76 (4.49–5.03)^b^	<0.001	5.00 (4.72–5.27)^b^	<0.001
Wearing mask	27,340 (91.36)	<0.001	5.80 (5.52–6.07)^b^	<0.001	6.06 (5.78–6.35)^b^	<0.001
Social distance	12,688 (42.40)	<0.001	2.01 (1.77–2.25)^b^	<0.001	2.47 (2.20–2.75)^b^	<0.001
**Awareness of COVID-19 pandemic**						
COVID-19 conspiracy beliefs		<0.001		<0.001		<0.001
Level 1	7,224 (24.14)		2.92 (2.53–3.31)^b^		3.06 (2.66–3.46)^b^	
Level 2	6,410 (21.42)		3.67 (3.21–4.13)^b^		3.76 (3.29–4.23)^b^	
Level 3	7,659 (25.59)		8.51 (7.89–9.14)^b^		9.31 (8.66–9.96)^b^	
Level 4	8,632 (28.85)		16.40 (15.62–17.19)^b^		15.47 (14.70–16.23)^b^	
Risk of COVID-19 infection		<0.001		<0.001		<0.001
Very high	2,188 (7.31)		11.38 (10.05–12.71)^b^		11.01 (9.70–12.33)^b^	
High	2,520 (8.42)		21.98 (20.37–23.6)^b^		20.32 (18.75–21.89)^b^	
Medium	4,636 (15.49)		14.75 (13.73–15.77)^b^		14.00 (13.00–15.00)^b^	
Low	15,107 (50.48)		4.41 (4.08–4.74)^b^		4.88 (4.54–5.22)^b^	
No	4,531 (15.14)		5.61 (4.94–6.28)^b^		5.52 (4.85–6.18)^b^	
Not sure	943 (3.15)		11.35 (9.32–13.37)^b^		12.83 (10.70–14.97)^b^	
Curability of COVID-19				<0.001		<0.001
Very high	12,611 (42.14)		3.61 (3.28–3.93)^b^		3.73 (3.40–4.06)^b^	
High	10,916 (36.48)		7.39 (6.90–7.88)^b^		7.20 (6.72–7.69)^b^	
Medium	3,625 (12.11)		21.32 (19.99–22.66)^b^		20.74 (19.42–22.06)^b^	
Low	1,529 (5.11)		20.21 (18.20–22.22)^b^		21.06 (19.02–23.10)^b^	
No	515 (1.72)		15.92 (12.76–19.08)^b^		17.28 (14.02–20.55)^b^	
Not sure	729 (2.44)		12.07 (9.71–14.44)^b^		12.48 (10.08–14.88)^b^	
**COVID-19 vaccine exception**						
Channel of vaccine information		<0.001		<0.001		<0.001
Community worker	8,416 (28.12)		4.62 (4.17–5.07)^b^		4.72 (4.26–5.17)^b^	
Internet	15,522 (51.87)		7.26 (6.85–7.67)^b^		7.40 (6.99–7.81)^b^	
Others	5,987 (20.01)		16.67 (15.73–17.61)^b^		16.10 (15.17–17.03)^b^	
Vaccine conspiracy beliefs		<0.001		<0.001		<0.001
Level 1	7,033 (23.50)		2.79 (2.40–3.17)^b^		2.70 (2.32–3.08)^b^	
Level 2	6,920 (23.12)		3.18 (2.77–3.59)^b^		3.41 (2.98–3.84)^b^	
Level 3	8,168 (27.29)		7.16 (6.60–7.72)^b^		7.44 (6.87–8.01)^b^	
Level 4	7,804 (26.08)		19.39 (18.51–20.26)^b^		18.91 (18.04–19.78)^b^	
Weigh risks of vaccination against risks of the disease		<0.001		<0.001		<0.001
Disease outweigh vaccine	18,862 (64.27)		5.48 (5.16–5.81)^b^		5.31 (4.99–5.63)^b^	
Vaccine outweigh disease	10,487 (35.73)		13.25 (12.61–13.90)^b^		13.44 (12.78–14.09)^b^	
Other life/health responsibilities		<0.001		<0.001		<0.001
Very high	14,607 (48.81)		2.88 (2.60–3.15)^b^		2.84 (2.57–3.11)^b^	
High	8,821 (29.48)		4.84 (4.39–5.29)^b^		5.76 (5.27–6.25)^b^	
Medium	3,656 (12.22)		22.73 (21.37–24.09)^b^		23.11 (21.75–24.48)^b^	
Low	1,796 (6.00)		32.96 (30.79–35.14)^b^		28.45 (26.37–30.54)^b^	
Very low	1,045 (3.49)		23.35 (20.78–25.91)^b^		22.11 (19.59–24.62)^b^	
Type of vaccination		<0.001		<0.001		<0.001
Unvaccinated	221 (0.74)		76.02 (70.39–81.65)^b^		79.19 (73.83–84.54)^b^	
Viral vector	3,737 (12.49)		6.50 (5.71–7.29)^b^		5.91 (5.16–6.67)^b^	
Inactivated	14,853 (49.63)		6.27 (5.88–6.66)^b^		6.32 (5.93–6.71)^b^	
Protein subunit	4,066 (13.59)		19.43 (18.21–20.65)^b^		19.16 (17.95–20.37)^b^	
Accept all vaccines	7,048 (23.55)		5.42 (4.89–5.95)^b^		5.62 (5.08–6.16)^b^	
Convenience of vaccination		<0.001		<0.001		<0.001
High	28,164 (94.14)		7.28 (6.98–7.59)^b^		7.34 (7.03–7.64)^b^	
Medium	1,401 (4.68)		25.34 (23.06–27.62)^b^		24.20 (21.95–26.44)^b^	
Low	360 (1.20)		30.00 (25.27–34.73)^b^		29.17 (24.47–33.86)^b^	
**Trust in health care system**						
Trust in doctors		<0.001		<0.001		<0.001
Level 1	8,502 (28.41)		18.22 (17.40–19.04)^b^		18.15 (17.33–18.97)^b^	
Level 2	7,344 (24.54)		7.94 (7.32–8.56)^b^		8.02 (7.40–8.64)^b^	
Level 3	7,092 (23.70)		4.09 (3.63–4.55)^b^		3.95 (3.49–4.40)^b^	
Level 4	6,987 (23.35)		1.32 (1.05–1.58)^b^		1.40 (1.13–1.68)^b^	
Trust in developers		<0.001		<0.001		<0.001
Level 1	8,100 (27.07)		19.85 (18.98–20.72)^b^		19.68 (18.81–20.54)^b^	
Level 2	10,145 (33.90)		7.37 (6.86–7.88)^b^		7.21 (6.70–7.71)^b^	
Level 3	11,680 (39.03)		1.35 (1.14–1.56)^b^		1.58 (1.36–1.81)^b^	

CI, confidence interval.

We categorized the score of COVID-19 conspiracy beliefs by quartiles as level 1 (≤7 points), level 2 (8–13 points), level 3 (14–20 points), and level 4 (≥21 points) and the score of vaccine conspiracy beliefs by quartiles as level 1 (≤7 points), level 2 (8–12 points), level 3 (13–18 points), and level 4 (≥19 points). We categorized the score of trust in doctors by quartiles as level 1 (≤30 points), level 2 (31–34 points), level 3 (35–38 points), and level 4 (≥39 points) and the score of trust in developers by quartiles as level 1 (≤17 points), level 2 (18–21 points), and level 3 (≥22 points).

a Differences between categories within each variable.

b Row percentages derived from the total number in the corresponding row.

c The mean ± standard deviation for variables.

Student’s t-tests for continuous variables and Chi-square tests for categorical variables.

### COVID-19 Vaccine Hesitancy and Its Influencing Factors

In the binary logistic regression model among all study participants, age, sex, educational level, marital status, self-report health condition, subjective social status in community level, smoking status, drinking status, healthy behaviors, the risk of COVID-19 infection, the curability of COVID-19, the channel of accessing information of COVID-19 vaccine, endorsement of vaccine conspiracy beliefs, weigh risks of vaccination against risks of the disease, other life/health responsibilities, inconvenience of vaccination, and lower trust in healthcare system were independent factors associated with hesitancy on COVID-19 primary vaccination. A similar pattern of hesitancy in the booster vaccination was also shown among the subjects (**Table 2**).

**Table 2:** Associations between COVID-19 vaccine hesitancy and sociodemographic, awareness of COVID-19 pandemic, COVID-19 vaccine exception, and trust in healthcare system.

Covariates	Primary vaccination				Booster vaccination			
	Model 1	*p*-value	Model 2	*p*-value	Model 1	*p*-value	Model 2	*p*-value
**Sociodemographic**								
Age (years)								
18–29	1.00 (ref.)		1.00 (ref.)		1.00 (ref.)		1.00 (ref.)	
30–39	0.59 (0.54–0.65)	<0.001	0.77 (0.68–0.88)	<0.001	0.58 (0.53–0.63)	<0.001	0.79 (0.70–0.90)	0.0002
40–49	0.40 (0.34–0.48)	<0.001	0.75 (0.60–0.93)	0.009	0.37 (0.31–0.44)	<0.001	0.66 (0.54–0.82)	0.0002
50–59	0.62 (0.49–0.79)	<0.001	0.89 (0.66–1.20)	0.451	0.67 (0.53–0.83)	0.0004	0.99 (0.75–1.32)	0.9683
60–	1.16 (0.81–1.65)	0.427	0.90 (0.55–1.46)	0.659	0.96 (0.65–1.41)	0.8292	0.68 (0.41–1.13)	0.1357
Sex								
Men	1.00 (ref.)		1.00 (ref.)		1.00 (ref.)		1.00 (ref.)	
Women	0.44 (0.40–0.48)	<0.001	0.80 (0.72–0.89)	<0.001	0.45 (0.42–0.49)	<0.001	0.79 (0.71–0.88)	<0.001
Educational status								
Below high school	1.00 (ref.)		1.00 (ref.)		1.00 (ref.)		1.00 (ref.)	
High school graduate	0.34 (0.30–0.38)	<0.001	0.69 (0.60–0.79)	<0.001	0.35 (0.31–0.39)	<0.001	0.67 (0.58–0.77)	<0.001
University graduate	0.22 (0.20–0.24)	<0.001	0.65 (0.56–0.74)	<0.001	0.26 (0.23–0.29)	<0.001	0.69 (0.60–0.79)	<0.001
Ethnic groups								
Han	1.00 (ref.)		1.00 (ref.)		1.00 (ref.)		1.00 (ref.)	
Minority	3.33 (2.91–3.82)	<0.001	1.07 (0.89–1.29)	0.4750	2.85 (2.47–3.28)	<0.001	0.95 (0.79–1.15)	0.5987
Religion								
Atheist	1.00 (ref.)		1.00 (ref.)		1.00 (ref.)		1.00 (ref.)	
Others	3.51 (3.21–3.84)	<0.001	1.19 (1.04–1.35)	0.0089	3.05 (2.79–3.34)	<0.001	1.08 (0.95–1.23)	0.2262
Marital status								
Married	1.00 (ref.)		1.00 (ref.)		1.00 (ref.)		1.00 (ref.)	
Others	1.91 (1.76–2.07)	<0.001	1.42 (1.27–1.58)	<0.001	1.95 (1.80–2.12)	<0.001	1.39 (1.25–1.56)	<0.001
Score of health condition	0.97 (0.96–0.97)	<0.001	1.00 (0.99–1.00)	0.0014	0.97 (0.96–0.97)	<0.001	1.00 (0.99–1.00)	0.0004
Subjective social status								
In China	1.09 (1.06–1.11)	<0.001	0.98 (0.94–1.02)	0.2301	1.06 (1.04–1.08)	<0.001	0.99 (0.95–1.03)	0.5260
In one’s community	1.10 (1.08–1.13)	<0.001	1.05 (1.01–1.09)	0.0173	1.07 (1.05–1.09)	<0.001	1.02 (0.98–1.06)	0.3521
Chronic condition								
0	1.00 (ref.)		1.00 (ref.)		1.00 (ref.)		1.00 (ref.)	
1	5.01 (4.54–5.53)	<0.001	1.57 (1.37–1.80)	<0.001	4.41 (3.99–4.88)	<0.001	1.53 (1.34–1.75)	<0.001
2	4.99 (4.22–5.89)	<0.001	1.08 (0.88–1.34)	0.4637	4.65 (3.93–5.49)	<0.001	1.24 (1.00–1.52)	0.0465
≥3	5.25 (3.97–6.94)	<0.001	0.90 (0.65–1.25)	0.5386	4.99 (3.77–6.59)	<0.001	1.03 (0.74–1.42)	0.8830
Smoking status								
Current smoker	1.00 (ref.)		1.00 (ref.)		1.00 (ref.)		1.00 (ref.)	
Former smoker	0.56 (0.47–0.66)	<0.001	1.12 (0.91–1.38)	0.2859	0.49 (0.41–0.58)	<0.001	0.86 (0.70–1.07)	0.1845
Never smoker	0.15 (0.14–0.16)	<0.001	0.61 (0.52–0.70)	<0.001	0.19 (0.17–0.21)	<0.001	0.71 (0.62–0.82)	<0.001
Drinking status								
Current drinker	1.00 (ref.)		1.00 (ref.)		1.00 (ref.)		1.00 (ref.)	
Former drinker	1.20 (1.00–1.44)	0.0508	1.55 (1.22–1.96)	0.0003	1.05 (0.87–1.27)	0.6256	1.23 (0.96–1.56)	0.0956
Never drinker	0.27 (0.24–0.30)	<0.001	0.92 (0.78–1.07)	0.2774	0.30 (0.27–0.34)	<0.001	0.88 (0.76–1.02)	0.1011
Health behaviors								
Washing hands								
Increased	1.00 (ref.)		1.00 (ref.)		1.00 (ref.)		1.00 (ref.)	
Unchanged/Decreased	5.18 (4.76–5.63)	<0.001	1.75 (1.57–1.97)	<0.001	5.18 (4.76–5.63)	<0.001	1.62 (1.45–1.81)	<0.001
Wearing mask								
Increased	1.00 (ref.)		1.00 (ref.)		1.00 (ref.)		1.00 (ref.)	
Unchanged/Decreased	9.12 (8.29–10.03)	<0.001	1.80 (1.58–2.04)	<0.001	7.62 (6.92–8.38)	<0.001	1.71 (1.50–1.94)	<0.001
Social distance								
Increased	1.00 (ref.)		1.00 (ref.)		1.00 (ref.)		1.00 (ref.)	
Unchanged/Decreased	7.35 (6.45–8.39)	<0.001	1.73 (1.48–2.03)	<0.001	5.75 (5.10–6.49)	<0.001	1.53 (1.32–1.77)	<0.001
**Awareness of COVID-19 pandemic**								
COVID-19 conspiracy beliefs								
Level 1	1.00 (ref.)		1.00 (ref.)		1.00 (ref.)		1.00 (ref.)	
Level 2	1.27 (1.05–1.53)	0.0148	0.99 (0.79–1.24)	0.9291	1.24 (1.03–1.49)	0.0243	0.93 (0.75–1.15)	0.5166
Level 3	3.09 (2.64–3.63)	<0.001	1.11 (0.89–1.38)	0.3567	3.25 (2.79–3.80)	<0.001	1.14 (0.93–1.41)	0.2122
Level 4	6.52 (5.62–7.57)	<0.001	1.19 (0.94–1.51)	0.1496	5.80 (5.01–6.71)	<0.001	1.05 (0.84–1.31)	0.6929
Risk of COVID-19 infection								
Very high	1.00 (ref.)		1.00 (ref.)		1.00 (ref.)		1.00 (ref.)	
High	2.19 (1.87–2.58)	<0.001	1.57 (1.29–1.91)	<0.001	2.06 (1.75–2.43)	<0.001	1.38 (1.13–1.68)	0.0013
Medium	1.35 (1.15–1.57)	0.0002	1.35 (1.11–1.63)	0.0025	1.32 (1.12–1.54)	0.0007	1.26 (1.04–1.53)	0.0168
Low	0.36 (0.31–0.42)	<0.001	0.93 (0.77–1.13)	0.4856	0.41 (0.36–0.48)	<0.001	0.98 (0.81–1.19)	0.8654
No	0.46 (0.39–0.56)	<0.001	1.02 (0.82–1.28)	0.8438	0.47 (0.39–0.57)	<0.001	0.98 (0.78–1.23)	0.8688
Not sure	1.00 (0.78–1.27)	0.9784	0.84 (0.59–1.20)	0.3311	1.19 (0.94–1.50)	0.1451	1.05 (0.75–1.46)	0.7910
Curability of COVID-19								
Very high	1.00 (ref.)		1.00 (ref.)		1.00 (ref.)		1.00 (ref.)	
High	2.13 (190–2.40)	<0.001	1.36 (1.19–1.56)	<0.001	2.00 (1.78–2.25)	<0.001	1.27 (1.11–1.46)	0.0004
Medium	7.24 (6.41–8.19)	<0.001	2.43 (2.09–2.82)	<0.001	6.76 (5.98–7.64)	<0.001	2.32 (2.00–2.68)	<0.001
Low	6.77 (5.79–7.91)	<0.001	2.82 (2.32–3.43)	<0.001	6.89 (5.91–8.04)	<0.001	3.01 (2.50–3.63)	<0.001
No	5.06 (3.93–6.52)	<0.001	2.01 (1.44–2.81)	<0.001	5.40 (4.22–6.90)	<0.001	2.57 (1.88–3.51)	<0.001
Not sure	3.67 (2.88–4.67)	<0.001	1.98 (1.42–2.77)	<0.001	3.68 (2.90–4.68)	<0.001	1.83 (1.32–2.54)	0.0003
**COVID-19 vaccine exception**								
Channel of vaccine information								
Community worker	1.00 (ref.)		1.00 (ref.)		1.00 (ref.)		1.00 (ref.)	
Internet	1.62 (1.44–1.82)	<0.001	1.39 (1.21–1.59)	<0.001	1.62 (1.44–1.82)	<0.001	1.36 (1.19–1.56)	<0.001
Others	4.13 (3.65–4.67)	<0.001	2.21 (1.90–2.56)	<0.001	3.88 (3.43–4.38)	<0.001	2.09 (1.81–2.42)	<0.001
Vaccine conspiracy beliefs								
Level 1	1.00 (ref.)		1.00 (ref.)		1.00 (ref.)		1.00 (ref.)	
Level 2	1.15 (0.94–1.39)	0.1734	1.07 (0.85–1.36)	0.5446	1.27 (1.05–1.54)	0.0152	1.32 (1.06–1.66)	0.0146
Level 3	2.69 (2.28–3.17)	<0.001	1.24 (1.00–1.55)	0.0507	2.90 (2.45–3.42)	<0.001	1.58 (1.27–1.95)	<0.001
Level 4	8.39 (7.20–9.77)	<0.001	1.37 (1.08–1.74)	0.0090	8.40 (7.20–9.81)	<0.001	1.77 (1.40–2.23)	<0.001
Weigh risks of vaccination against risks of the disease								
Disease outweigh vaccine	1.00 (ref.)		1.00 (ref.)		1.00 (ref.)		1.00 (ref.)	
Vaccine outweigh disease	2.64 (2.42–2.87)	<0.001	1.19 (1.07–1.32)	0.0009	2.77 (2.54–3.02)	<0.001	1.33 (1.20–1.47)	<0.001
Other life/health responsibilities								
Very high	1.00 (ref.)		1.00 (ref.)		1.00 (ref.)		1.00 (ref.)	
High	1.72 (1.50–1.97)	<0.001	0.96 (0.82–1.13)	0.6023	2.09 (1.83–2.39)	<0.001	1.16 (0.99–1.35)	0.0598
Medium	9.94 (8.78–11.25)	<0.001	2.14 (1.82–2.51)	<0.001	10.28 (9.08–11.64)	<0.001	2.26 (1.93–2.65)	<0.001
Low	16.61 (14.47–19.07)	<0.001	2.94 (2.48–3.49)	<0.001	13.60 (11.80–15.67)	<0.001	2.41 (2.02–2.86)	<0.001
Very low	10.29 (8.65–12.23)	<0.001	3.16 (2.57–3.90)	<0.001	9.71 (8.14–11.57)	<0.001	3.04 (2.46–3.75)	<0.001
Type of vaccination								
Unvaccinated	1.00 (ref.)				1.00 (ref.)			
Viral vector	0.02 (0.02–0.03)	<0.001	0.03 (0.02–0.04)	<0.001	0.02 (0.01–0.02)	<0.001	0.02 (0.02–0.04)	<0.001
Inactivated	0.02 (0.02–0.03)	<0.001	0.03 (0.02–0.05)	<0.001	0.02 (0.01–0.03)	<0.001	0.03 (0.02–0.05)	<0.001
Protein subunit	0.08 (0.06–0.11)	<0.001	0.08 (0.05–0.12)	<0.001	0.06 (0.05–0.09)	<0.001	0.08 (0.05–0.12)	<0.001
Accept all vaccines	0.02 (0.01–0.03)	<0.001	0.04 (0.03–0.06)	<0.001	0.02 (0.01–0.02)	<0.001	0.04 (0.02–0.06)	<0.001
Convenient vaccination								
High	1.00 (ref.)		1.00 (ref.)		1.00 (ref.)		1.00 (ref.)	
Medium	4.32 (3.80–4.91)	<0.001	1.56 (1.32–1.85)	<0.001	4.03 (3.54–4.59)	<0.001	1.42 (1.20–1.67)	<0.001
Low	5.46 (4.34–6.87)	<0.001	2.21 (1.63–3.00)	<0.001	5.20 (4.13–6.56)	<0.001	1.85 (1.37–2.50)	<0.001
**Trust in healthcare system**								
Trust in doctors								
Level 1	1.00 (ref.)		1.00 (ref.)		1.00 (ref.)		1.00 (ref.)	
Level 2	0.39 (0.35–0.43)	<0.001	0.79 (0.69–0.91)	0.0012	0.39 (0.36–0.44)	<0.001	0.80 (0.69–0.91)	0.0009
Level 3	0.19 (0.17–0.22)	<0.001	0.70 (0.57–0.84)	0.0002	0.19 (0.16–0.21)	<0.001	0.65 (0.54–0.78)	<0.001
Level 4	0.06 (0.05–0.07)	<0.001	0.67 (0.50–0.89)	0.0056	0.06 (0.05–0.08)	<0.001	0.68 (0.52–0.89)	0.0045
Trust in developers								
Level 1	1.00 (ref.)		1.00 (ref.)		1.00 (ref.)		1.00 (ref.)	
Level 2	0.32 (0.29–0.35)	<0.001	0.65 (0.57–0.74)	<0.001	0.32 (0.29–0.35)	<0.001	0.62 (0.55–0.71)	<0.001
Level 3	0.06 (0.05–0.07)	<0.001	0.45 (0.35–0.58)	<0.001	0.07 (0.06–0.08)	<0.001	0.49 (0.39–0.62)	<0.001

We categorized the score of COVID-19 conspiracy beliefs by quartiles as level 1 (≤7 points), level 2 (8–13 points), level 3 (14–20 points), and level 4 (≥21 points) and the score of vaccine conspiracy beliefs by quartiles as level 1 (≤7 points), level 2 (8–12 points), level 3 (13–18 points), and level 4 (≥19 points). We categorized the score of trust in doctors by quartiles as level 1 (≤30 points), level 2 (31–34 points), level 3 (35–38 points), and level 4 (≥39 points) and the score of trust in developers by quartiles as level 1 (≤17 points), level 2 (18–21 points), and level 3 (≥22 points).

Model 1: unadjusted.

Model 2: adjusted age, sex, educational status, ethnic groups, religion, marital status, change one’s job, family doctor, score of health condition, subjective social status in China, subjective social status in one’s community, body mass index, chronic condition, smoking status, drinking status, health behaviors, COVID-19 conspiracy beliefs, risk of COVID-19 infection, curability of COVID-19, channel of vaccine information, vaccine conspiracy beliefs, weigh risks of vaccination against risks of the disease, other life/health responsibilities, trust in doctors, trust in developers, and convenient vaccination.

### Variation of COVID-19 Vaccine Hesitancy

Our study highlighted that the prevalence of vaccine hesitancy among Chinese residents remains at a low and stable level, with a slight shift from 8.40% to 8.39% (95% CI, 8.07 to 8.70) that was observed between the primary and booster vaccinations. In detail, 88.02% and 4.81% of the population responded in a consistent acceptance and hesitancy towards taking a COVID-19 vaccine in both phases, respectively. Notably, there were also individuals who showed varying levels of willingness to take vaccines. In summary, 3.58% of respondents declared an acceptance of rejection, whereas 3.59% of those who were previously hesitant became willing to receive vaccination (**Table 3**). The associations of COVID-19 vaccine hesitancy transformations and socio-demographic, awareness of COVID-19 pandemic, COVID-19 vaccine exception, and trust in healthcare system were summarized in **Table 3**. The variation of willingness (i.e., acceptance to hesitancy, hesitancy to acceptance, hesitancy to hesitancy) to take a COVID-19 vaccine was associated with age, sex, educational level, marital status, chronic disease condition, smoking, healthy behaviors, the curability of COVID-19, the channel of accessing information of COVID-19 vaccine, endorsement of vaccine conspiracy beliefs, weigh risks of vaccination against risks of the disease, other life/health responsibilities, and lower trust in healthcare system were independent.

**Table 3:** Associations between COVID-19 vaccine hesitancy transformations and sociodemographic, awareness of COVID-19 pandemic, COVID-19 vaccine exception, and trust in healthcare system.

Covariates	Acceptance to hesitancy		Hesitancy to acceptance		Hesitancy to hesitancy	
	Model 1	Model 2	Model 1	Model 2	Model 1	Model 2
**Sociodemographic**						
Age (years)						
18–29	1.00 (ref.)	1.00 (ref.)	1.00 (ref.)	1.00 (ref.)	1.00 (ref.)	1.00 (ref.)
30–39	0.60 (0.52–0.68)^*^	0.72 (0.61–0.86)^*^	0.63 (0.56–0.72)^*^	0.73 (0.61–0.87)^*^	0.54 (0.48–0.61)^*^	0.75 (0.64–0.89)^*^
40–49	0.34 (0.26–0.45)^*^	0.51 (0.37–0.70)^*^	0.41 (0.32–0.53)^*^	0.65 (0.48–0.89)^*^	0.37 (0.30–0.47)^*^	0.72 (0.54–0.96)^*^
50–59	0.49 (0.33–0.72)^*^	0.74 (0.47–1.14)	0.39 (0.26–0.61)^*^	0.60 (0.37–0.99)^*^	0.76 (0.58–1.00)^*^	1.08 (0.76–1.55)
60–	0.68 (0.35–1.33)	0.64 (0.30–1.39)	1.09 (0.63–1.87)	0.97 (0.50–1.88)	1.17 (0.75–1.84)	0.79 (0.41–1.52)
Sex						
Men	1.00 (ref.)	1.00 (ref.)	1.00 (ref.)	1.00 (ref.)	1.00 (ref.)	1.00 (ref.)
Women	0.48 (0.42–0.55)^*^	0.77 (0.66–0.90)^*^	0.45 (0.40–0.51)^*^	0.78 (0.67–0.91)^*^	0.41 (0.37–0.46)^*^	0.74 (0.64–0.85)^*^
Educational status						
Below high school	1.00 (ref.)	1.00 (ref.)	1.00 (ref.)	1.00 (ref.)	1.00 (ref.)	1.00 (ref.)
High school graduate	0.35 (0.29–0.41)^*^	0.62 (0.51–0.77)^*^	0.33 (0.28–0.39)^*^	0.66 (0.54–0.80)^*^	0.30 (0.26–0.35)^*^	0.64 (0.53–0.77)^*^
University graduate	0.29 (0.25–0.33)^*^	0.70 (0.58–0.85)^*^	0.21 (0.18–0.24)^*^	0.63 (0.52–0.77)^*^	0.20 (0.18–0.23)^*^	0.58 (0.48–0.70)^*^
Ethnic groups						
Han	1.00 (ref.)	1.00 (ref.)	1.00 (ref.)	1.00 (ref.)	1.00 (ref.)	1.00 (ref.)
Minority	2.62 (2.10–3.26)^*^	1.00 (0.76–1.31)	3.60 (2.96–4.37)^*^	1.13 (0.87–1.46)^*^	3.49 (2.93–4.15)^*^	0.99 (0.77–1.26)
Religion						
Atheist	1.00 (ref.)	1.00 (ref.)	1.00 (ref.)	1.00 (ref.)	1.00 (ref.)	1.00 (ref.)
Others	3.02 (2.64–3.46)^*^	1.17 (0.98–1.39)	3.99 (3.51–4.55)^*^	1.38 (1.15–1.64)^*^	3.54 (3.16–3.98)^*^	1.18 (1.00–1.40)
Marital status						
Married	1.00 (ref.)	1.00 (ref.)	1.00 (ref.)	1.00 (ref.)	1.00 (ref.)	1.00 (ref.)
Others	1.72 (1.52–1.94)^*^	1.22 (1.04–1.43)^*^	1.64 (1.45–1.85)^*^	1.22 (1.04–1.44)*	2.22 (2.00–2.47)^*^	1.63 (1.40–1.88)^*^
Score of health condition	0.97 (0.96–0.97)^*^	0.99 (0.99–1.00)	0.97 (0.96–0.97)^*^	0.99 (0.99–1.00)^*^	0.96 (0.96–0.97)^*^	1.00 (0.99–1.00)^*^
Subjective social status						
In China	1.09 (1.06–1.12)^*^	1.09 (1.05–1.12)^*^	1.15 (1.11–1.18)^*^	0.99 (0.94–1.05)	1.05 (1.02–1.08)^*^	0.97 (0.92–1.02)
In one’s community	0.99 (0.99–1.00)^*^	0.99 (0.99–1.00)^*^	1.17 (1.13–1.20)^*^	1.07 (1.01–1.13)^*^	1.07 (1.04–1.09)^*^	1.03 (0.98–1.08)
Chronic condition						
0	1.00 (ref.)	1.00 (ref.)	1.00 (ref.)	1.00 (ref.)	1.00 (ref.)	1.00 (ref.)
1	3.76 (3.23–4.38)^*^	1.46 (1.20–1.77)^*^	4.90 (4.23–5.68)^*^	1.47 (1.21–1.77)^*^	5.78 (5.10–6.55)^*^	1.99 (1.67–2.38)^*^
2	5.06 (3.99–6.41)^*^	1.37 (1.03–1.82)^*^	5.83 (4.61–7.37)^*^	1.08 (0.81–1.44)	5.35 (4.31–6.64)^*^	1.22 (0.92–1.62)
≥3	5.26 (3.53–7.83)^*^	1.04 (0.67–1.63)	5.86 (3.93–8.74)^*^	0.81 (0.52–1.27)	5.89 (4.13–8.40)^*^	0.94 (0.61–1.45)
Smoking status						
Current smoker	1.00 (ref.)	1.00 (ref.)	1.00 (ref.)	1.00 (ref.)	1.00 (ref.)	1.00 (ref.)
Former smoker	0.36 (0.27–0.49)^*^	0.72 (0.51–1.01)	0.51 (0.40–0.65)^*^	1.13 (0.85–1.50)	0.54 (0.44–0.67)^*^	1.16 (0.88–1.52)
Never smoker	0.20 (0.18–0.23)^*^	0.72 (0.59–0.88)^*^	0.12 (0.11–0.14)^*^	0.52 (0.42–0.64)^*^	0.15 (0.14–0.17)^*^	0.63 (0.52–0.77)^*^
Drinking status						
Current drinker	1.00 (ref.)	1.00 (ref.)	1.00 (ref.)	1.00 (ref.)	1.00 (ref.)	1.00 (ref.)
Former drinker	0.78 (0.56–1.08)	1.04 (0.72–1.50)	1.09 (0.83–1.44)	1.62 (1.16–2.25)^*^	1.26 (1.00–1.59)	1.62 (1.19–2.22)^*^
Never drinker	0.31 (0.26–0.36)^*^	0.77 (0.62–0.95)^*^	0.23 (0.19–0.28)^*^	0.85 (0.66–1.08)	0.28 (0.24–0.32)^*^	0.94 (0.76–1.15)
Health behaviors						
Washing hands						
Increased	1.00 (ref.)	1.00 (ref.)	1.00 (ref.)	1.00 (ref.)	1.00 (ref.)	1.00 (ref.)
Unchanged/Decreased	4.06 (3.58–4.60)^*^	1.40 (1.19–1.64)^*^	5.05 (4.46–5.71)^*^	1.57 (1.33–1.85)^*^	7.31 (6.54–8.15)^*^	2.07 (1.79–2.40)^*^
Wearing mask						
Increased	1.00 (ref.)	1.00 (ref.)	1.00 (ref.)	1.00 (ref.)	1.00 (ref.)	1.00 (ref.)
Unchanged/Decreased	6.72 (5.81–7.78)^*^	1.60 (1.33–1.93)^*^	9.40 (8.19–10.78)^*^	1.82 (1.52–2.19)^*^	11.75 (10.44–13.24)^*^	2.14 (1.82–2.52)^*^
Social distance						
Increased	1.00 (ref.)	1.00 (ref.)	1.00 (ref.)	1.00 (ref.)	1.00 (ref.)	1.00 (ref.)
Unchanged/Decreased	5.08 (4.28–6.02)^*^	1.58 (1.30–1.93)^*^	8.69 (7.05–10.71)^*^	2.12 (1.67–2.70)^*^	7.13 (6.03–8.43)^*^	1.49 (1.22–1.82)^*^
**Awareness of COVID-19 pandemic**						
COVID-19 conspiracy beliefs						
Level 1	1.00 (ref.)	1.00 (ref.)	1.00 (ref.)	1.00 (ref.)	1.00 (ref.)	1.00 (ref.)
Level 2	1.44 (1.09–1.90)^*^	1.01 (0.75–1.36)	1.53 (1.15–2.04)*	1.22 (0.88–1.69)	1.11 (0.86–1.42)	0.89 (0.66–1.21)
Level 3	3.32 (2.62–4.20)*	1.07 (0.80–1.44)	2.96 (2.29–3.81)*	1.09 (0.78–1.52)	3.33 (2.73–4.07)*	1.36 (1.03–1.80)*
Level 4	6.40 (5.12–8.00)*	0.93 (0.68–1.28)	8.21 (6.51–10.36)*	1.23 (0.86–1.75)	6.20 (5.13–7.50)*	1.21 (0.89–1.65)
Risk of COVID-19 infection						
Very high	1.00 (ref.)	1.00 (ref.)	1.00 (ref.)	1.00 (ref.)	1.00 (ref.)	1.00 (ref.)
High	1.97 (1.57–2.48)^*^	1.42 (1.09–1.85)^*^	2.19 (1.76–2.73)^*^	1.72 (1.33–2.22)^*^	2.48 (1.98–3.10)^*^	1.60 (1.21–2.10)^*^
Medium	0.96 (0.77–1.21)	1.10 (0.85–1.43)	1.04 (0.83–1.29)	1.19 (0.92–1.54)	1.68 (1.36–2.08)^*^	1.54 (1.18–2.01)^*^
Low	0.36 (0.29–0.44)^*^	0.89 (0.69–1.16)	0.26 (0.21–0.33)^*^	0.79 (0.61–1.03)	0.43 (0.35–0.54)^*^	1.07 (0.82–1.40)
No	0.35 (0.27–0.46)^*^	0.84 (0.61–1.14)	0.34 (0.26–0.45)^*^	0.88 (0.64–1.21)	0.56 (0.43–0.71)^*^	1.11 (0.81–1.51)
Not sure	0.83 (0.58–1.18)	0.94 (0.58–1.50)	0.53 (0.35–0.79)^*^	0.55 (0.31–0.97)^*^	1.48 (1.10–2.00)^*^	0.83 (0.53–1.31)
Curability of COVID-19						
Very high	1.00 (ref.)	1.00 (ref.)	1.00 (ref.)	1.00 (ref.)	1.00 (ref.)	1.00 (ref.)
High	1.90 (1.61–2.24)^*^	1.30 (1.09–1.56)^*^	2.14 (1.81–2.53)^*^	1.47 (1.22–1.78)^*^	2.20 (1.87–2.58)^*^	1.35 (1.12–1.63)^*^
Medium	5.67 (4.75–6.77)^*^	2.17 (1.77–2.67)^*^	6.50 (5.44–7.77)^*^	2.38 (1.92–2.95)^*^	9.22 (7.83–10.85)^*^	2.97 (2.44–3.62)^*^
Low	5.26 (4.17–6.62)^*^	2.85 (2.18–3.72)^*^	4.98 (3.91–6.35)^*^	2.51 (1.88–3.36)^*^	9.54 (7.84–11.62)^*^	4.03 (3.15–5.17)^*^
No	3.63 (2.44–5.40)^*^	2.02 (1.27–3.21)^*^	2.94 (1.87–4.60)^*^	1.11 (0.62–1.99)	7.57 (5.60–10.22)^*^	2.97 (1.99–4.43)^*^
Not sure	3.01 (2.10–4.31)^*^	1.95 (1.23–3.08)^*^	2.94 (2.02–4.28)^*^	2.20 (1.36–3.56)^*^	4.63 (3.41–6.28)^*^	1.88 (1.22–2.90)^*^
**COVID-19 vaccine exception**						
Channel of vaccine information						
Community worker	1.00 (ref.)	1.00 (ref.)	1.00 (ref.)	1.00 (ref.)	1.00 (ref.)	1.00 (ref.)
Internet	1.52 (1.28–1.79)^*^	1.34 (1.12–1.61)^*^	1.51 (1.28–1.80)^*^	1.40 (1.15–1.69)^*^	1.75 (1.49–2.05)^*^	1.54 (1.28–1.86)^*^
Others	3.15 (2.63–3.76)^*^	1.99 (1.62–2.44)^*^	3.59 (3.01–4.30)^*^	2.15 (1.75–2.66)^*^	5.02 (4.26–5.91)^*^	2.69 (2.20–3.28)^*^
Vaccine conspiracy beliefs						
Level 1	1.00 (ref.)	1.00 (ref.)	1.00 (ref.)	1.00 (ref.)	1.00 (ref.)	1.00 (ref.)
Level 2	1.44 (1.07–1.93)^*^	1.52 (1.10–2.09)^*^	1.13 (0.83–1.53)	1.09 (0.78–1.53)	1.17 (0.91–1.50)	1.16 (0.85–1.57)
Level 3	2.86 (2.20–3.72)^*^	1.79 (1.31–2.43)^*^	2.39 (1.84–3.09)^*^	1.16 (0.84–1.60)	3.00 (2.43–3.71)^*^	1.51 (1.13–2.00)^*^
Level 4	10.51 (8.26–13.39)^*^	2.29 (1.63–3.21)^*^	10.28 (8.15–12.98)^*^	1.41 (1.00–2.00)	8.55 (7.01–10.42)^*^	1.36 (0.99–1.85)
Weigh risks of vaccination against risks of the disease						
Disease outweigh vaccine	1.00 (ref.)	1.00 (ref.)	1.00 (ref.)	1.00 (ref.)	1.00 (ref.)	1.00 (ref.)
Vaccine outweigh disease	2.82 (2.48–3.19)^*^	1.25 (1.08–1.44)^*^	2.53 (2.24–2.87)^*^	1.04 (0.89–1.20)	2.93 (2.63–3.27)^*^	1.28 (1.12–1.46)^*^
Other life/health responsibilities						
Very high	1.00 (ref.)	1.00 (ref.)	1.00 (ref.)	1.00 (ref.)	1.00 (ref.)	1.00 (ref.)
High	2.20 (1.83–2.64)^*^	1.25 (1.02–1.54)^*^	1.48 (1.21–1.81)^*^	0.87 (0.69–1.09)	2.02 (1.67–2.43)^*^	1.05 (0.85–1.31)
Medium	8.80 (7.35–10.52)^*^	2.04 (1.63–2.55)^*^	8.21 (6.86–9.83)^*^	1.77 (1.41–2.22)^*^	13.93 (11.80–16.45)^*^	2.70 (2.18–3.34)^*^
Low	12.01 (9.74–14.81)^*^	2.19 (1.71–2.80)^*^	16.97 (14.00–20.56)^*^	2.89 (2.29–3.65)^*^	21.57 (17.94–25.95)^*^	3.24 (2.57–4.08)^*^
Very low	9.24 (7.17–11.91)^*^	2.85 (2.12–3.84)^*^	10.24 (8.03–13.06)^*^	3.09 (2.32–4.12)^*^	12.81 (10.18–16.11)^*^	3.88 (2.92–5.15)^*^
Type of vaccination						
Unvaccinated	1.00 (ref.)	1.00 (ref.)	1.00 (ref.)	1.00 (ref.)	1.00 (ref.)	1.00 (ref.)
Viral vector	0.05 (0.03–0.09)^*^	0.11 (0.05–0.23)^*^	0.09 (0.05–0.16)^*^	0.24 (0.10–0.57)^*^	0.01 (0.00–0.01)^*^	0.01 (0.00–0.01)^*^
Inactivated	0.05 (0.03–0.09)^*^	0.15 (0.07–0.30)^*^	0.08 (0.04–0.14)^*^	0.27 (0.12–0.63)^*^	0.01 (0.00–0.01)^*^	0.01 (0.01–0.02)^*^
Protein subunit	0.14 (0.08–0.24)^*^	0.29 (0.15–0.60)^*^	0.21 (0.11–0.39)^*^	0.54 (0.23–1.25)	0.03 (0.02–0.05)^*^	0.04 (0.02–0.06)^*^
Accept all vaccines	0.03 (0.02–0.05)^*^	0.13 (0.06–0.26)^*^	0.04 (0.02–0.08)^*^	0.22 (0.09–0.52)^*^	0.01 (0.01–0.01)^*^	0.02 (0.01–0.03)^*^
Convenient vaccination						
High	1.00 (ref.)	1.00 (ref.)	1.00 (ref.)	1.00 (ref.)	1.00 (ref.)	1.00 (ref.)
Medium	3.03 (2.46–3.75)^*^	1.21 (0.94–1.55)	3.55 (2.91–4.33)^*^	1.41 (1.10–1.81)^*^	5.52 (4.73–6.45)^*^	1.71 (1.39–2.11)^*^
Low	3.36 (2.26–5.01)^*^	1.47 (0.90–2.38)	3.78 (2.58–5.54)^*^	2.07 (1.31–3.27)^*^	7.66 (5.87–9.99)^*^	2.81 (1.95–4.06)^*^
**Healthcare system**						
Trust in doctors						
Level 1	1.00 (ref.)	1.00 (ref.)	1.00 (ref.)	1.00 (ref.)	1.00 (ref.)	1.00 (ref.)
Level 2	0.40 (0.34–0.46)^*^	0.78 (0.64–0.94)^*^	0.38 (0.33–0.44)^*^	0.78 (0.64–0.95)^*^	0.35 (0.31–0.40)^*^	0.78 (0.65–0.94)^*^
Level 3	0.17 (0.14–0.21)^*^	0.52 (0.40–0.69)^*^	0.18 (0.15–0.22)^*^	0.58 (0.44–0.77)^*^	0.17 (0.15–0.20)^*^	0.70 (0.54–0.91)^*^
Level 4	0.07 (0.06–0.10)^*^	0.56 (0.39–0.82)^*^	0.06 (0.05–0.09)^*^	0.55 (0.36–0.83)^*^	0.05 (0.04–0.07)^*^	0.72 (0.49–1.06)
Trust in developers						
Level 1	1.00 (ref.)	1.00 (ref.)	1.00 (ref.)	1.00 (ref.)	1.00 (ref.)	1.00 (ref.)
Level 2	0.30 (0.26–0.34)^*^	0.66 (0.55–0.79)^*^	0.31 (0.27–0.36)^*^	0.71 (0.59–0.87)^*^	0.29 (0.26–0.33)^*^	0.58 (0.49–0.69)^*^
Level 3	0.08 (0.07–0.10)^*^	0.68 (0.49–0.93)^*^	0.06 (0.05–0.08)^*^	0.61 (0.43–0.87)^*^	0.04 (0.03–0.05)^*^	0.36 (0.26–0.51)^*^

^*^p < 0.05.

We categorized the score of COVID-19 conspiracy beliefs by quartiles as level 1 (≤7 points), level 2 (8–13 points), level 3 (14–20 points), and level 4 (≥21 points) and the score of vaccine conspiracy beliefs by quartiles as level 1 (≤7 points), level 2 (8–12 points), level 3 (13–18 points), and level 4 (≥19 points). We categorized the score of trust in doctors by quartiles as level 1 (≤30 points), level 2 (31–34 points), level 3 (35–38 points), and level 4 (≥39 points) and the score of trust in developers by quartiles as level 1 (≤17 points), level 2 (18–21 points), and level 3 (≥22 points).

Model 1: unadjusted.

Model 2: adjusted age, sex, educational status, ethnic groups, religion, marital status, change one’s job, family doctor, score of health condition, subjective social status in China, subjective social status in one’s community, body mass index, chronic condition, smoking status, drinking status, health behaviors, COVID-19 conspiracy beliefs, risk of COVID-19 infection, curability of COVID-19, channel of vaccine information, vaccine conspiracy beliefs, weigh risks of vaccination against risks of the disease, other life/health responsibilities, trust in doctors, trust in developers, and convenient vaccination.

### Sensitivity Analysis

In sensitivity analyses, exclusion of cases with chronic disease did not appreciably alter the findings for vaccine hesitancy. The effect estimates remained similar for the main results (**Appendix Table 2**).

## Discussion

The current study examined the prevalence of COVID-19 vaccine hesitancy in a large representative sample of 31 provinces of mainland China. Our findings indicated that a sizable majority (88.02%) of mainland Chinese citizens express their readiness to be vaccinated. It is possible that this proportion will remain robust throughout the upcoming COVID-19 vaccine booster, but this is likely to be researched and confirmed. Our predicted vaccine hesitancy rate is comparable with earlier research conducted on the majority of the Chinese residents. According to these surveys, the vaccination rate among Chinese residents was found to be around 80%. The reason for the lower vaccination hesitancy rate, or for the greater vaccine acceptance rate, is mostly attributable to the following factors: Firstly, China has established a vaccine management law and successfully passed the World Health Organization’s evaluation of its National Vaccine Regulatory System (NRS) which guarantees its quality and supply of the vaccine (22, 23). Secondly, China has consistently enhanced postmarket surveillance of vaccinations, with an emphasis on the safety and effectiveness of the vaccine while making a consistent follow-up on the incidences of vaccine-preventable disease as well as public acceptance of the vaccines. In addition, tracking the experience of vaccine use together with the development of vaccine big data are still ongoing (24). Thirdly, China has strengthened risk communication to ensure that recipients and the public have a consciousness of the benefits and risks of vaccination and to actively disseminate the scientific concept that the overall benefits of the vaccination greatly outweigh the risks. Finally, China has engaged in expanding vaccine availability, which requires vaccination services to be tailored to the characteristics of the jurisdiction area and population, as well as a reasonable distribution of vaccination clinics.

At the provincial level, the prevalence of COVID-19 vaccine hesitation varies greatly. Our results show that among the 31 provinces, the prevalence of vaccine hesitancy was more than 10% in three provinces. The reason for this level of hesitancy is not yet clear, and a variety of factors may be involved. Since the first vaccine was approved for marketing in mainland China on December 31, 2020, various provinces have made strenuous efforts to increase primary vaccination rates, but there are significant differences in the demographic structure, health literacy, prevalence of chronic diseases, and vaccination service supply capacity among provinces. It is likely that the combination of these factors has led to the uneven distribution of vaccine hesitation rates among provinces. With 4.81% of the population refusing to receive a COVID-19 vaccination, in the current study, the timeline for eradicating the pandemic may be delayed, resulting in widespread of vaccine hesitancy, wreaking havoc on individuals and the healthcare systems. Thus, in the future, policy development in China should prioritize minimizing existing inequalities among provinces when it comes to vaccination.

COVID-19 vaccine hesitancy increases with inconvenience of vaccination. Although China established tens of thousands of temporary vaccination sites in a relatively short time, vaccination service is provided through appointments and the waiting time at the vaccination site is frequently longer due to limited health personnel resources and a shortage of vaccines. This situation brings a lot of inconvenience to vaccinators and may have played a significant role towards vaccine acceptance rate. In order to effectively prevent the spread of the virus, vaccinators have to be registered through a reliable and user-friendly appointment system. Additionally, the majority of residents in China are not yet accustomed to vaccine appointments, which creates a considerable “sense of inconvenience” for vaccinators, which in turn causes some people to have doubts about whether to receive a vaccination. Therefore, for improving COVID-19 vaccination uptake, it is particularly important to improve the experience of vaccination services. Key measures that should also be considered include increasing the number of vaccination personnel, vaccine supply, encouraging qualified medical institutions to provide vaccination services, and actively using digital technologies to reduce waiting time (25–27).

There is a considerable link between doctor and vaccine developer distrust and COVID-19 vaccine hesitation. In essence, willingness to take a vaccination is a matter of trust: that the vaccine is necessary, that it will function, and that it is safe. Due to recent vaccination-related adverse events and instances of counterfeit vaccine, the public’s trust in medical professionals and vaccine developers has decreased significantly (28). To build faith in the vaccine, the vaccination service organization should, on the one hand, expand the number of vaccination medical personnel, train and develop doctor-patient communication skills, and improve the quality of vaccination service evaluation. China should also accelerate the development of a vaccine industry credibility system, encourage vaccine production, and encourage companies to take the lead in vaccine production and circulation while ensuring the quality and safety of vaccine products from development stage to circulation. In addition, recommending the one vaccine in which at the given moment is with the highest level of the public willingness will likely result in a less prevalence of COVID-19 vaccination hesitancy.

It is worth mentioning that when the role of gender in COVID-19 vaccine hesitancy was assessed, males were shown to be more likely to reject the vaccine. The finding was consistent with a previous research in which a higher vaccine acceptance rate was associated with men’s increased perception of COVID-19 vaccine and decreased belief in disease-related conspiracy theories (11). However, our research was carried out at the stage when the vaccination rate had already exceeded 60%. As information about the COVID-19 vaccination circulated, women may have become fully aware of the implications of the disease and so lost belief in conspiratorial claims, implying that their vaccine hesitancy rate was found to be lower than that of males. In the future COVID-19 vaccination, attention should be paid to increasing the vaccination rate of men. Additionally, age, men, educational level, marital status, self-report health condition, subjective social status, smoking status, healthy behaviors, the curability of COVID-19, the channel of accessing information of COVID-19 vaccine, endorsement of vaccine conspiracy beliefs, weigh risks of vaccination against risks of the disease, and other life/health responsibilities were all found to be independently associated with COVID-19 vaccine hesitancy. Numerous reports show that the mechanism underlying vaccine hesitancy is exceedingly complicated, and that effective countermeasures should be implemented concurrently (17, 20, 29, 30). Firstly, we think that assisting persons with poor information and insufficient health literacy in obtaining a correct understanding of vaccines through education may play a critical role. Government authorities should also communicate clearly and consistently in order to instill public confidence in vaccination programs. This involves describing how vaccines function and are created, from recruiting to regulatory approval based on safety and efficacy. Effective campaigns should also carefully describe the level of effectiveness of a vaccine, the duration of protection (with multiple doses if necessary), and the critical nature of population-wide coverage in order to attain the herd immunity. Secondly, vaccine information transmitted by the Internet and the media should be effectively identified and any misleading information must be eliminated. The Internet and other forms of media should serve as a link between vaccination services and the general population through disseminating vaccine knowledge received through official channels and eradicating social misconceptions about the vaccines.

### Strengths and Weaknesses of the Study

This is the first large-scale study to assess the prevalence and associated factors of COVID-19 vaccination hesitancy in a large, saturated sample of the Chinese population. Due to the saturation of the sample, we can be certain that our estimate of vaccine hesitancy is accurate. To provide more extensive explanatory variables, we adopted the most widely accepted international definition of vaccine hesitancy and collected data using the EAH and 3C frameworks. One of the major limitations of the current study is that it relies on self-reports of willingness to take a COVID-19 vaccination to assess vaccine hesitancy, and we were unable to develop a standard for validation due to the lack of a universal scale to assess COVID-19 vaccine hesitancy in China. Due to the fact that an accurate assessment of COVID-19 vaccine hesitancy can serve as an important basis for vaccine development and production, as well as the estimation of market demand, the development of a global scale for COVID-19 vaccine hesitancy assessment will become one of the important directions of future research. However, the COVID-19 vaccine hesitancy was assessed from a reliable questionnaire and the results of it was similar with previous studies according to the Oxford COVID-19 Vaccine Hesitancy Scale (13, 31). Another shortcoming of the study includes its cross-sectional design, which precluded the establishment of a cause-and-effect link. Finally, despite the fact that we used data from a large saturation sample of the population from 31 provinces, due to the epidemic, we were forced to collect data *via* online questionnaires utilizing the snowball sampling approach. Therefore, these research findings may differ from those estimated using probability sampling. In addition, the influence of socioeconomic level on COVID-19 vaccination hesitancy observed in this study may not be applicable to persons without Internet access.

## Conclusions

Despite the aforementioned constraints, COVID-19 vaccination hesitancy prevalence in China is modest in comparison with other countries. This will lay a solid foundation for future booster vaccinations. However, interprovincial disparities in COVID-19 vaccine hesitation may delay the onset of herd immunity, and local vaccination efforts should be stepped up in Tianjin, Hebei, Beijing, and Hainan provinces due to their significantly greater frequency of COVID-19 vaccine hesitancy. Emphasis should be placed on building trust in medical personnel and vaccine producers, promoting the convenience of vaccination services, and spreading reliable COVID-19 vaccine information *via* the Internet and other media.

## Data Availability Statement

The raw data supporting the conclusions of this article will be made available by the authors, without undue reservation.

## Author Contributions

Conceptualization: YM, JW, QL, MW, and JG. Data curation: YM, QL, WW, and MM. Formal analysis: JW, QL, and YM. Funding acquisition: MW and JG. Investigation: JW, YM, QL, MM, LZ, and ZM. Methodology: YM, JW, QL, and CT. Project administration: JW. Resources: JW and YM. Software: QL, LZ, and ZM. Writing—original draft: JW, YM, QL, and CT. Writing—review and editing: YM, CT, MW, and JG. All authors contributed to the article and approved the submitted version.

## Funding

This study is supported by the National Social Science Fund of China (number 21BGL222); the Collaborative Innovation Key Project of Zhengzhou (number 20XTZX05015); Joint Project of National Health Commission and Henan Province (number SB201901072); 2021 Postgraduate Education Reform and Quality Improvement Project of Henan Province (number YJS2021KC07); Performance Evaluation of New Basic Public Health Service Projects in Henan Province (number 2020130B).

## Conflict of Interest

The authors declare that the research was conducted in the absence of any commercial or financial relationships that could be construed as a potential conflict of interest.

## Publisher’s Note

All claims expressed in this article are solely those of the authors and do not necessarily represent those of their affiliated organizations, or those of the publisher, the editors and the reviewers. Any product that may be evaluated in this article, or claim that may be made by its manufacturer, is not guaranteed or endorsed by the publisher.
