# Supplementary material for: COVID-19 Vaccine Hesitancy Among Chinese Population: A Large-Scale National Study
Source: Front Immunol. 2021 Nov 29;12:781161. doi: 10.3389/fimmu.2021.781161 (PMC8666422; doi:10.3389/fimmu.2021.781161)
Supplement: Supplementary file 1 [file DataSheet_1.docx]

**Appendix Fig. 1 The trend of prevalence of COVID-19 vaccine hesitancy over sample saturation.**


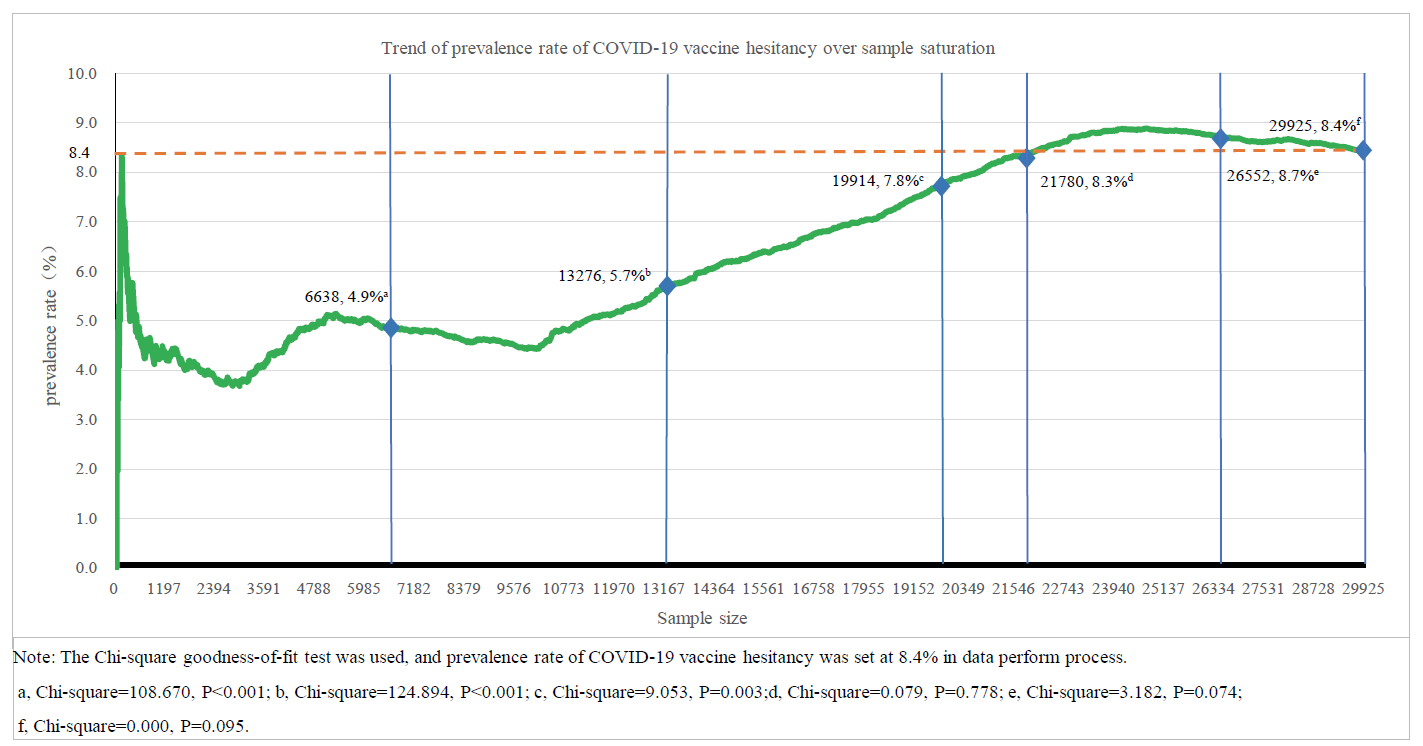


Note: The Chi-square goodness-of-fit test was used;

Red dotted line was the prevalence of COVID-19 vaccine hesitancy in our study (8.40%)

a, Chi-square=108.670, *P*<0.001; b, Chi-square=124.894, *P*<0.001; c, Chi-square=9.053, *P*=0.003; d, Chi-square=0.079, *P*=0.778; e, Chi-square=3.182, *P*=0.074; f, Chi-square=0.000, *P*=0.095.

**Appendix 1 Data collection and explanatory variables.**

# Section 1. Personal information questionnaire

# I. General Information

| 1. The name |
| --- |
| 1. What's your gender？ A. male B. female |
| 1. How old are you？ Age |
| 1. What's your height in centimeters？ cm（centimeter）,How many kilograms do you weigh？ kg（kilogram） |
| 1. What is your nationality？ National |
| 1. What is your religion？ 2. Not religious B. Buddhism C. Christian D. Taoism E. Islam F. Other |
| 1. What is your marital status?   A. Unmarried B. Married C. Divorced D. Death of a spouse E. Other |
| 1. What is your education level?   A. Illiteracy B. Primary and below C. Junior high school D. Senior High School (Technical secondary school) E. University and above |
| 1. What is your occupation type？   A. Workers in transportation, construction, production, manufacturing and other industries  B. Personnel in agriculture, forestry, animal husbandry, fishing, water conservancy and other industries (farmers, fishermen, etc.)  C. Business or service workers (self-employed, service workers, etc.)  D. Students  E. Professional and technical personnel (medical workers, accountants, teachers, scientific researchers, etc.)  F. Persons in charge of state organs, party and mass organizations, enterprises and institutions  F. Retire/leave  G. Unfixed occupation  H. Other |
| 1. Has COVID-19 changed your work？   A. No  B. No, but I changed my place of work, eg working from home  C. Yes, my working hours have been reduced  D. Yes, my working hours have increased  E. Yes, I am unemployed now  F Yes, I have a new full-time job  G. Yes, I have a new part-time job |
| 1. Which of the following insurance policies do you have? (multiple choices)   A. New rural cooperative medical insurance B. Basic medical insurance for urban workers C. Basic medical insurance for urban residents D. Basic medical insurance for urban and rural residents E. Miho unity F. Commercial insurance G. without cover H. Other |
| 1. Are you under contract with a family doctor？   A .Yes B. No C. Don't know |
| 1. The number of times you left your city of residence in the last three months？ _____ Frequency |
| 1. How many people are there in your family？ People，What was the total family income last year？_____ Ten thousand yuan |
| 1. Who are the family members you live with？（multiple choices）   A. spouse B. Children C. Parents D. Son-in-law/daughter-in-law E. Parents-in-law  F. Grandparents G. Grandchildren H. Brothers and sisters I. Living alone J. Other |
| 1. Do you have any children attending school now？ A. Yes B.No C. No children |
| 1. Do you currently suffer from a medically diagnosed chronic disease?（multiple choices）   A. No B. Hypertension C. Diabetes D. Stroke E. Coronary heart disease (CHD) F. Chronic obstructive pulmonary disease G. Cancer H. Other |
| 1. Have you been tested for nucleic acid？   A. Yes, negative B. Yes, positive results C. No, but YES D. No, and I won't |

# II．Lifestyle behavior

| **（一）Healthy lifestyle** |
| --- |
| 1. Do you maintain a reasonable weight？   A. Always B. Often C. Sometimes D. Little E. Never |
| 1. Do you control unhealthy diet such as high salt and fat？   A. Always B. Often C. Sometimes D. Little E. Never |
| 1. How do you participate in light or moderate intensity physical activity? Such as walking, square dancing, etc)   A. Always B. Often C. Sometimes D. Little E. Never |
| 1. Do you get enough？   A. Always B. Often C. Sometimes D. Little E. Never |
| Do you smoke (including e-cigarettes)？  A. Always B. Often C. Sometimes D. Little E. Have to give up smoking F. Never smoking |
| 1. Do you drink alcohol？   A. Always B. Often C. Sometimes D. Little E. Have to stop drinking F. Never drank |
| 1. Do you pay attention to psychological decompression? Yoga, singing, holidays, etc)   A. Always B. Often C. Sometimes D. Little E. Never |
| 1. How do you relate to people around you？   A. Very good B. Good C. General D. No good E. Very bad |
| 1. Are you constantly studying or working？   A. Always B. Often C. Sometimes D. Little E. Never |
| 1. Do you have regular health check-ups (excluding those for illness)？   A. Always B. Often C. Sometimes D. Little E. Never |
| **（二）COVID-19 transmission interruption behavior** |
| 1. Do you wash your hands more often than before COVID-19？   A. Significantly increased B. Significantly increased C. No significant change D. Decreased E. Significantly decreased |
| 1. Do you use hand sanitizer (soap) more often than before COVID-19？   A. Significantly increased B. Significantly increased C. No significant change D. Decreased E. Significantly decreased |
| 1. Are you wearing masks more often than you did before COVID-19？   A. Significantly increased B. Significantly increased C. No significant change D. Decreased E. Significantly decreased |
| 1. Do you participate in gathering activities more frequently than before COVID-19？   A. Significantly increased B. Significantly increased C. No significant change D. Decreased E. Significantly decreased |
| 1. Do you spend any more time at home than before COVID-19？   A. Significantly increased B. Significantly increased C. No significant change D. Decreased E. Significantly decreased |
| 1. Have you consciously increased social distancing (distance of more than 1m between people) compared to pre-COVID-19？   A. Always B. Often C. Sometimes D. Little E. Never |

# III. Quality of life Assessment (EQ-5D)

| 1. Do you have any difficulty getting around today？   A. No difficulty B. A little bit difficult C. Moderate difficulty D. Be in serious difficulty E. Unable to get around |
| --- |
| 1. Did you have trouble doing everyday activities like washing or dressing today？   A. No difficulty B. A little bit difficult C. Moderate difficulty D. Be in serious difficulty E. Unable to bathe or dress themselves |
| 1. Do you have any difficulty in your work, study, housework, leisure activities today？   A. No difficulty B. A little bit difficult C. Moderate difficulty D. Be in serious difficulty E. Inability to carry out daily activities |
| 1. Do you feel any pain or discomfort today？   A. No pain B. A little bit sore C. Moderate pain or discomfort D. Severe pain or discomfort E. Very severe pain or discomfort |
| 1. Are you feeling anxious or depressed today？   A. No anxiety or depression B. A little anxiety or depression C. Moderate anxiety or depression D. Have severe anxiety or depression E. Very severe anxiety or depression |
| 1. If 0 is the worst and 100 is the best, how would you rate your health today？   \|-----\|—---\|—---\|—---\|—---\|—---\|—---\|—---\|—---\|—---\|  0 10 20 30 40 50 60 70 80 90 100  Please write the number you marked on the scale in the space below.  Your health today = points |

# IV. Community environmental assessment

| 1. Is it convenient to get vaccinated against COVID-19？   A. Very convenient B. Convenient C. General D. Inconvenient E. Very inconvenient |
| --- |
| 1. Do you have the same views on COVID-19 vaccination as those around you？   A. Exactly the same B. The same C. Not sure D. Not the same E. Totally different |
| 1. Are you willing to discuss COVID-19 vaccine issues with people around you？   A. Very satisfied B. Satisfied C. Not so satisfied D. Dissatisfied E. Very dissatisfied |
| 1. Do you think it is necessary to set up special vaccination sites for COVID-19？   A. Very necessary B. Necessary C. Not sure D. Not necessary E. Totally unnecessary |
| 1. What do you think of the performance of local leaders in COVID-19 vaccination？   A. Very good B. Good C. General D. Bad E. Very bad |
| 1. Do you care what people around you think of you？   A. Very care B. Care C General D. Don't care E. Don't care a bit |
| 1. Do people around you care and help each other？   A. Always B. Often C. Sometimes D. Little E. Never |
| 1. Do you want to live here for a long time？   A. Very hope B. Hope C. General D. Don't hope E. Very don't hope |

# V. General vaccination knowledge - letter - line assessment

**General vaccines refer to all vaccines other than COVID-19 vaccines, including category I and II vaccines: hepatitis B vaccine, BCG vaccine, DPT vaccine, influenza vaccine, HPV vaccine, rabies vaccine, etc.**

| **（一）General vaccination awareness** |
| --- |
| 1. Diseases that are normally controlled by vaccines can be treated with antibiotics.   A. Correct B. Incorrect C. Unclear |
| 1. Infectious diseases such as chickenpox and smallpox were eradicated by the widespread use of general vaccines.   A. Correct B. Incorrect C. Unclear |
| 1. The efficacy of general vaccines has been proven.   A. Correct B. Incorrect C. Unclear |
| 1. Regular vaccination can improve children's resistance.   A. Correct B. Incorrect C. Unclear |
| 1. Common vaccinations can cause diseases such as diabetes, autism and multiple sclerosis.   A. Correct B. Incorrect C. Unclear |
| 1. Multiple doses of common vaccines can damage a child's immune system.   A. Correct B. Incorrect C. Unclear |
| 1. The development of a child's immune system is stunted by ordinary vaccines.   A. Correct B. Incorrect C. Unclear |
| 1. Normal doses of vaccines are not dangerous to humans.   A. Correct B. Incorrect C. Unclear |
| 1. Regular vaccinations increase the incidence of allergies.   A. Correct B. Incorrect C. Unclear |
| **（二）General vaccination beliefs** |
| 1. Are you concerned about the safety risks of general vaccinations？   A .Very worry B. Worry C. General D. Not worry E. Very unconcerned |
| 1. Are you worried about the effectiveness of regular vaccinations？   A .Very worry B. Worry C. General D. Not worry E. Very unconcerned |
| **（三）General vaccination behavior** |
| 1. Have you been vaccinated before？   A. Yes B. No**（skip to 6,1）** C. Unclear |
| 1. What information do you pay attention to when choosing a general vaccine？   A. Security B. Effectiveness C. Price D. Vaccination way E. Inoculum time F. Do not pay attention to, mainly rely on the recommendation of medical staff G. Other |
| 1. Have you had any adverse reactions during the course of general vaccination？   A .Yes B. No**（skip to 6,1）** C. Unclear |
| 1. What are your adverse reactions？   A. Local reaction: pain, induration, mass, rash, pruritus, etc  B. Systemic reaction: fatigue, fever, diarrhea, constipation, dysphagia, loss of appetite, vomiting, pain, dyspnea, etc  C. Other |

# VI. COVID-19 progress assessment

| 1. At present, do you think the Novel Coronavirus has mutated？   A .Yes B. No C. unclear |
| --- |
| 1. Currently, how many people do you think are infected with COVID-19 globally？   A. More than 200 million B. 100 million - 200 million C. 50 million – 100 million D. 50 million the following E. Unclear |
| 1. Do you think the COVID-19 pandemic is serious？   A. Very serious B. Serious C. General D. Not too serious E. Nothing serious at all F. Unclear |
| 1. Is the COVID-19 outbreak serious in your place of residence？   A. Very serious B. Serious C. General D. Not too serious E. Nothing serious at all F. Unclear |
| 1. In the future, do you think the COVID-19 epidemic will continue or rebound in your place？   A. Highly possible B. Possible C. Not sure D. Impossible E. Absolutely impossible F. Unclear |
| 1. Currently, do you think you are at high risk of contracting COVID-19？   A. Very high risk B. High risk C. In the risk D. Low risk E. No risk F. Unclear |
| 1. Currently, do you think it is possible to cure COVID-19？   A. Highly possible B. Possible C. Not sure D. Impossible E. Absolutely impossible F. Unclear |

# Section 2 COVID-19 vaccination Status Questionnaire

# VII. COVID-19 vaccine information

| （一）Sources of COVID-19 vaccine information |
| --- |
| 1. What are the main sources of information about COVID-19 vaccines that you have obtained since the outbreak？   A. Community workers (e.g. street office staff, village head, etc.)  B. Internet (wechat group, wechat official account, Douyin, Kuaishou, Weibo, etc.)  C. Family/relatives/friends/neighbors, etc D. TV E. Medical staff F. Newspaper, magazine, leaflet, etc G. Lectures, symposiums or seminars related to COVID-19 vaccines H. Other |
| 1. What do you think is the most reliable way to get information on COVID-19 vaccines since the outbreak？   A. Community workers (e.g. street office staff, village head, etc.)  B. Internet (wechat group, wechat official account, Douyin, Kuaishou, Weibo, etc.)  C. Family/relatives/friends/neighbors, etc D. TV E. Medical staff F. Newspaper, magazine, leaflet, etc G. Lectures, symposiums or seminars related to COVID-19 vaccines H. Other |
| （二）COVID-19 vaccine awareness |
| 1. Do you think vaccination against COVID-19 is effective？   A. Very effective B. Effective C. Not sure D. Invalid E. Completely invalid F. Unclear |
| 1. Do you think there will be shock, breathing difficulties and other serious adverse reactions after receiving COVID-19 vaccine？   A. Must B. May C. Not sure D. May not E. Not completely F. Unclear |
| 1. How long do you think the protection period of COVID-19 vaccine is？   A. Less than 1 month B. 1-3 months C. 3-6 months D. 6-month-1 year E. More than 1 year F. Unclear |

# VIII. Willingness to be vaccinated against COVID-19

| 1. Are you looking forward to getting vaccinated against COVID-19 before it becomes available？   A. Very hope B. Hope C. General D. Don't hope E. Very don't hope |
| --- |
| 1. Do you want to be vaccinated against COVID-19 in the early days？   A. Yes and already vaccinated B. Yes, but not at that time C. Hesitation and delay D. Those refusing E. Refuse vaccination and convince people around you not to |
| 1. Do you want to be vaccinated against COVID-19？   A. Yes and already vaccinated B. Yes, but not at that time C. Hesitation and delay D. Those refusing E. Refuse vaccination and convince people around you not to |
| 1. Are you willing to be vaccinated regularly if you need to be？   A. Yes and already vaccinated B. Yes, but not at that time C. Hesitation and delay D. Those refusing E. Refuse vaccination and convince people around you not to |
| 1. Which COVID-19 vaccine would you prefer to get at present？   A. One shot (Adenovirus vector vaccine) B. Two injections (Inactivated vaccine） C. Triple Dose (Recombinant Novel Coronavirus Vaccine) D. Above all can E. Reluctance to vaccinate |
| 1. Would you like to recommend COVID-19 vaccination to your family/friends？   A. Very willing B. Willing C. General D. Unwillingness E. Very reluctant |
| 1. At present, do you think it is necessary to vaccinate everyone against COVID-19？   A. Strongly necessary B. Be necessary C. Not sure D. Not necessary E. Totally unnecessary |
| 1. In the future, do you think it is necessary for all staff to have regular vaccination against COVID-19？   A. Strongly necessary B. Be necessary C. Not sure D. Not necessary E. Totally unnecessary |

# IX. COVID-19 vaccination practices

| 1. Currently, how many people around you are estimated to have been vaccinated against COVID-19？   A. Below 30% B. 30%-50% C. 50%-80% D. Above 80% E. 100% F. Unclear |
| --- |
| 1. Have you completed the first round of COVID-19 vaccination？   A .Have been inoculated. B. Are vaccinated C .No, but get ready for vaccination**（skip to 14）**  D. No, I'm not sure yet**（skip to 15）** E. No, and I don't plan**（skip to 16）** |
| 1. Have you deliberately waited for people around you to get vaccinated before making an appointment？   A. Yes B. No**（skip to 5）** |
| 1. What was the main reason you decided to get vaccinated later than others？   A. Vaccine safety concerns B. Concerns about vaccine effectiveness C. Doubts about the vaccine D. To vaccinate those in greater need, such as health care workers E. Have confidence in resisting COVID-19 infection F. believe the outbreak will soon end F. Other reasons |
| 1. How do you book COVID-19 vaccines？   A. Community (village) committee unified arrangement B. Online booking C. Telephone booking D. On-site registration E. Other |
| 1. Which institution did you get your COVID-19 vaccine at？   A. Temporary inoculation site B. Community-level medical and health institutions C. The centers for disease control and prevention D. Hospitals at county level and above  E. Other |
| 1. One of the biggest reasons to get vaccinated against COVID-19 is？   A. Prevention of COVID - 19 B. Free C. The influence of people around you D. Community (village) committees require vaccination E. Recommended by professionals (medical staff, medical specialists, etc. F. Work demand G. Convenient life and travel H. Don't want to wear a mask I. Get rewards J .Accidental factors K. Fear of a long-term outbreak |
| 1. What kind of COVID-19 vaccine did you get？   A. One shot (Adenovirus vector vaccine) B. Two doses (Inactivated vaccine) C. Triple Dose (Recombinant Novel Coronavirus Vaccine) D. Unclear |
| 1. What is the main reason for you to get the COVID-19 vaccine？   A. Vaccination site availability B. Higher security C. More effective D. Vaccination cycles are short E. Recommended by medical staff F. more convenient |
| 1. What do you think are the differences between different types of COVID-19 vaccines？   A. Different security B. Different validity C. Different doses of inoculation D. Different protection cycles E. No difference F. Other |
| 1. Have you experienced any adverse reactions after receiving COVID-19 vaccine？   A. Yes B. No C. Unclear |
| 1. Do you have any doubts after COVID-19 vaccination？   A .Very doubt B. Doubt C. General D. No doubt**（skip to 10,1）** E. No doubt at all**（skip to 10,1）** |
| 1. What is the most important reason for your doubts after COVID-19 vaccination？**（skip to 10,1）**   A. Vaccine safety B. Vaccine effectiveness C. The vaccine is doubtful D. Short protection period E. Other |
| 1. What is the main reason you haven't been vaccinated yet？**（skip to 14,1）**   A. Inoculation sites are far away B. Booking less than C. There is no vaccine available at the vaccination site D. The appointment is not available  E. Not eligible for inoculation F. Vaccine safety concerns G. Concerns about vaccine effectiveness H. Need to know more about vaccines I. The vaccine is doubtful G. Short protection period K. Influence of people around you L. Too much time between stitches M. Low risk of infection N. Have confidence in resisting COVID-19 infection O. believe the outbreak will soon end |
| 1. What is the most important reason you are not sure about getting vaccinated against COVID-19？**（skip to 14,1）**   A. Vaccine safety concerns B. Concerns about vaccine effectiveness C. Need to know more about vaccines D. The vaccine is doubtful E. Short protection period F. Influence of people around you G. Too much time between stitches H. Low risk of infection I. Have confidence in resisting COVID-19 infection G. Believe the outbreak will soon end |
| 1. What is one of the main reasons you are not going to get vaccinated against COVID-19？**（skip to 14,1）**   A .Vaccine safety concerns B. Concerns about vaccine effectiveness C. Need to know more about vaccines D. The vaccine is doubtful E. Short protection period F. Influence of people around you G. Too much time between stitches H. Low risk of infection I. Have confidence in resisting COVID-19 infection G. believe the outbreak will soon end |

# X. COVID-19 vaccination service experience

| 1. COVID-19 vaccination makes you anxious？   A. Very anxious B. Anxious C. General D. Not anxious E. Not at all |
| --- |
| 1. You feel excited about getting vaccinated against COVID-19？   A. Very excited B. Excited C. General D. Not excited E. Not at all |
| 1. You feel relieved to get vaccinated against COVID-19？   A. Very easy B. Easy C. General D. Not easy E. Not at all |
| 1. COVID-19 vaccination makes you angry？   A. Very angry B. Angry C. General D. Not angry E. Not at all |
| 1. COVID-19 vaccination makes you happy？   A. Very happy B. Happy C. General D. Not happy E. Very unhappy |
| 1. Getting vaccinated against COVID-19 makes you sick？   A .Very tired B. Tired C. General D. Not boring E. Not at all |
| 1. Getting vaccinated against COVID-19 makes you feel humiliated？   A. Very disgraceful B. Disgraceful C. General D. Graceful E. Very graceful |
| 1. When you ask medical staff about COVID-19 vaccines, do you understand their answers？   A. Often B. Sometimes C. May not D. Unclear |
| 1. Before COVID-19 vaccination, medical staff at the COVID-19 vaccination site can identify whether you meet the requirements for vaccination (such as fever, antibiotics, etc.)？   A. Yes B. No |
| 1. Have the medical staff at the vaccination site explained to you the purpose of vaccination against COVID-19？   A. Yes B. No |
| 1. Do you want to be able to choose the type or dose of COVID-19 vaccine？   A. Yes, absolutely B. Yes, to a certain extent C. Doesn't matter |
| 1. Did the medical staff at the vaccination site explain in detail the possible adverse reactions (such as redness and pain at the vaccination site)?   A .Yes B. No |
| 1. Whether the medical staff at the COVID-19 vaccination site prompts you to stay for observation？   A. Yes B. No |
| 1. 14. Will the medical staff at the vaccination site inform you of the precautions after vaccination (such as drinking more water and keeping water away from the vaccination site)?   A .Yes B .No |
| 1. In general, were you treated with respect and dignity at the vaccination site？   A. Yes, quite often B .Yes, occasionally C. No |
| 1. Are you willing to receive a second round of COVID-19 vaccination if necessary？   A. Very willing B. Willing C. General D. Unwillingness E. Very reluctant F. Unclear |

# XI. Satisfaction with COVID-19 vaccination service

| 1.Are you satisfied with the waiting time at the vaccination point?  A .Very satisfied B. Satisfied C. Not so satisfied D. Dissatisfied E. Very dissatisfied |
| --- |
| 2.Are you satisfied with the medical staff's attention to your health condition?  A .Very satisfied B. Satisfied C. Not so satisfied D. Dissatisfied E. Very dissatisfied |
| 3.Are you satisfied with the distance between the vaccination site and your place of residence?  A .Very satisfied B. Satisfied C. Not so satisfied D. Dissatisfied E. Very dissatisfied |
| 4.Are you satisfied with the environment and hygiene of COVID-19 vaccination sites?  A .Very satisfied B. Satisfied C. Not so satisfied D. Dissatisfied E. Very dissatisfied |
| 5.Are you satisfied with the whole process of COVID-19 vaccination?  A .Very satisfied B. Satisfied C. Not so satisfied D. Dissatisfied E. Very dissatisfied |

# XII. Accessibility of COVID-19 vaccination services

| 1.How far are you from the nearest COVID-19 vaccination site?  A. Less than 0.5 km B. 0.5 to 1 km C. 1-3 km D. 3-5 km E. Greater than 5 km  F. Unclear (skip to 13, 4) |
| --- |
| 2.What is your mode of transportation to the COVID-19 vaccination site?  A. Walk B. Ride an electric bike C. Ride a motorcycle D. Drive a car E. Take public transport (bus, subway, etc.) F. Others |
| 3.How long does it take you to get to the nearest COVID-19 vaccination site?  A. Less than 15 minutes B. 15-30 minutes C. 30-60 minutes D. Greater than 1 hour |

# XIII. Health economics evaluation of COVID-19 vaccination services

| 1.How long do you have to wait in line to get your COVID-19 vaccine? Minutes |
| --- |
| 2.How much do you need to travel (including oil) to and from the COVID-19 vaccination site? (RMB) |
| 3.How much salary will you lose (time/piece rate, overtime, full attendance bonus, etc.) due to COVID-19 vaccination? (RMB) |
| 1. Assuming COVID-19 vaccination costs money, how much would you be willing to pay for yourself?   A. <100 yuan B. 101-201 yuan C. 201-300 yuan D. 301-400 yuan E. ≥400 yuan  F. Refused vaccination at his own expense |

# Section 3. Special evaluation questionnaire

**XIV.Subjective social status scale**

1.At the top of the ladder are the richest -- they have the most money, the most education, the most respected jobs.

At the bottom of the ladder are the worst off -- those with the least money, the least education, the least respected jobs or no jobs at all.

The higher up the ladder you are, the closer you are to the people at the top; The lower you go, the closer you are to the people at the bottom.

**Please mark the steps with an "X"** (0 at the bottom, 10 at the top) where you think you are (relative to other Chinese citizens).
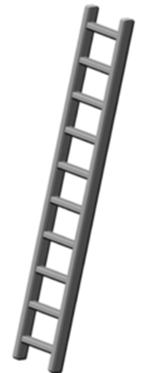


2. People define community in different ways; Please define it in whatever way makes most sense to you.

At the top of the ladder are the highest ranking people in the community.

At the bottom of the ladder are the lowest ranking people in the community.

Ask you to mark the ladder with an "X" (0 at the bottom, 10 at the top) where you think you are (relative to others in your community).

**XV. Skepticism about COVID-19**

| 1.Novel Coronavirus is a hoax.  A. Strongly agree B. Agree C. Generally D. Disagree E. Strongly disagree F. Don't know |
| --- |
| 2.The novel coronavirus is artificially produced  A. Strongly agree B. Agree C. Generally D. Disagree E. Strongly disagree F. Don't know |
| 3.The novel coronavirus transmission is designed to reduce the size of the global population.  A. Strongly agree B. Agree C. Generally D. Disagree E. Strongly disagree F. Don't know |
| 4.A group of powerful people deliberately spread the novel Coronavirus in order to make money.  A. Strongly agree B. Agree C. Generally D. Disagree E. Strongly disagree F. Don't know |
| 5.Some governments have deliberately transmitted novel Coronavirus in order to gain political control.  A. Strongly agree B. Agree C. Generally D. Disagree E. Strongly disagree F. Don't know |
| 6.Some countries deliberately transmit the novel coronavirus in order to destabilize other countries.  A. Strongly agree B. Agree C. Generally D. Disagree E. Strongly disagree F. Don't know |
| 7.Certain transnational corporations deliberately spread novel coronavirus in order to gain control.  A. Strongly agree B. Agree C. Generally D. Disagree E. Strongly disagree F. Don't know |

**XVI.COVID-19 Vaccine Skepticism**

| 1.Inaccurate COVID-19 vaccine safety data.  A. Strongly agree B. Agree C. Generally D. Disagree E. Strongly disagree F. Don't know |
| --- |
| 2.COVID-19 vaccinations are harmful, but the message is being buried.  A. Strongly agree B. Agree C. Generally D. Disagree E. Strongly disagree F. Don't know |
| 3.Pharmaceutical companies covered up the dangers of COVID-19 vaccines.  A. Strongly agree B. Agree C. Generally D. Disagree E. Strongly disagree F. Don't know |
| 4.People are being lied about the effectiveness of COVID-19 vaccines.  A. Strongly agree B. Agree C. Generally D. Disagree E. Strongly disagree F. Don't know |
| 5.Inaccurate COVID-19 vaccine effectiveness data.  A. Strongly agree B. Agree C. Generally D. Disagree E. Strongly disagree F. Don't know |
| 6.People are being lied about the safety of COVID-19 vaccines.  A. Strongly agree B. Agree C. Generally D. Disagree E. Strongly disagree F. Don't know |
| 7.COVID-19 vaccination can cause other diseases.  A. Strongly agree B. Agree C. Generally D. Disagree E. Strongly disagree F. Don't know |

**XVII.Doctors and Vaccine Developers Scale**

**For each question, please select your level of agreement**

|  | **Strongly agree** | **Agree** | **General** | **Disagree** | **Strongly disagree** |
| --- | --- | --- | --- | --- | --- |
| (1) Medical personnel |  |  |  |  |  |
| 1. I trust the medical staff |  |  |  |  |  |
| 2.The medical staff didn't really care about me |  |  |  |  |  |
| 3.The medical staff have my best interests at heart |  |  |  |  |  |
| 4. Medical staff don't respect me |  |  |  |  |  |
| 5. The medical staff want to do their best |  |  |  |  |  |
| 6. The medical staff didn't understand my living situation |  |  |  |  |  |
| 7. Medical staff often make mistakes |  |  |  |  |  |
| 8.Medical professionals choose this profession for the money |  |  |  |  |  |
| 9.The medical staff will give me the vaccine even if it's bad for me |  |  |  |  |  |
| (2) Vaccine developers |  |  |  |  |  |
| 1.Vaccine developers put vaccine safety first |  |  |  |  |  |
| 2. I don't trust vaccine developers |  |  |  |  |  |
| 3.Vaccine developers just want to make money |  |  |  |  |  |
| 4.Vaccine developers don't care enough to help others |  |  |  |  |  |
| 5.Vaccine developers fail to properly check vaccine safety |  |  |  |  |  |

**XVIII.COVID-19 Vaccine Hesitancy Scale**

**For each question, please select your level of agreement**

|  | **Strongly agree** | **Agree** | **General** | **Disagree** | **Strongly disagree** |
| --- | --- | --- | --- | --- | --- |
| 1.COVID-19 vaccines are essential to one's health |  |  |  |  |  |
| 2. The coronavirus vaccine is effective |  |  |  |  |  |
| 3. Getting vaccinated against COVID-19 is important for the health of those around you |  |  |  |  |  |
| 4.All COVID-19 vaccines that the government plans to provide are beneficial |  |  |  |  |  |
| 5. New vaccines are riskier than old ones |  |  |  |  |  |
| 6.The information I received about COVID-19 vaccines was reliable and credible |  |  |  |  |  |
| 7.Vaccination is an effective way to avoid COVID-19 infection |  |  |  |  |  |
| 8.People should follow the advice of the medical staff |  |  |  |  |  |
| 9.I am concerned about severe adverse reactions to COVID-19 vaccines |  |  |  |  |  |

**Section 4 Guardian's willingness to vaccinate adolescents against COVID-19**

| 1. How many teenagers are there in your family?  A. None(The end of the answer) B. One C. Two D. Three or more |
| --- |
| 2.Did you delay giving them the free vaccines provided by the state? (e.g. DPT vaccine, hepatitis B vaccine, BCG vaccine, poliomyelitis vaccine, etc.)  A.Yes B.No C.Unclear |
| 3.Do you refuse to vaccinate them with the free vaccines provided by the state? (e.g. DPT vaccine, hepatitis B vaccine, BCG vaccine, poliomyelitis vaccine, etc.)  A.Yes B.No C.Unclear |
| 4. Do you think it is important for their health to be vaccinated?  A Very important B. Important C.Unclear D. Unimportance E.Not at all important |
| 5.Are you willing to have them vaccinated against COVID-19?  A.Yes (jump to 7 questions) B.Not sure but inclined to vaccinate (jump to 8 questions) C.Not sure but inclined not to vaccinate D.Unwilling |
| 6. What is one of the main reasons you don't want them to be vaccinated against COVID-19? (Skip to Question 9)  A.Is concerned about the vaccine's safety B.Is concerned about its effectiveness C.The child is too young D.The risk of infection is low  E.Inoculation is too troublesome F.Influenced by people around G. Others |
| 7.Have they been vaccinated against COVID-19?  A. Yes B. No C. Uncertain |
| 8. What is one of the main reasons you would like them to be vaccinated against COVID-19?  A.Prevention of COVID-19 B.Free C.Impact of people around D.Vaccination required by community (village) committees  E.Professionals (medical staff, medical experts, etc.) recommend that F.Needs G.To live and travel easily to go to school  H.Not wearing a mask I. Can get a reward I accidental factors |
| 9. Assuming COVID-19 vaccination costs money, how much would you be willing to pay?  A. <100 yuan B.101-201 yuan C.201-300 yuan D.301-400 yuan E.≥400 yuan  F.Refused vaccination at his own expense |

Adolescents in this study refer to those between the ages of 3 and 18; Guardians refer to the adults who actually bear the responsibility of juvenile guardianship during the implementation of the investigation. Such as: teenagers' parents, grandparents, etc.; In the title, "they" refers specifically to the teenagers in the respondents' families.

**Appendix Fig. 2** the prevalence of vaccine hesitancy in all age groups by sex.

**Appendix Table 1 The Age- and sex-****standardized prevalence of COVID-19 vaccine hesitancy by province.**

| **Province** | **Hesitancy rate in the first phase of vaccination** | | **Predicted hesitancy rate in the second phase of vaccination** | |
| --- | --- | --- | --- | --- |
|  | Crude (%) | Age- and sex-standardized (%) | Crude (%) | Age- and sex-standardized (%) |
| Anhui | 4.19(2.44-5.95) | 3.87(2.53-5.87) | 6.39(4.25-8.53) | 5.9(4.19-8.25) |
| Beijing | 13.04(11.82-14.26) | 11.82(10.72-13.01) | 11.27(10.12-12.41) | 10.15(9.13-11.26) |
| Chongqing | 6.52(2.95-10.09) | 5.95(3.39-10.23) | 6.52(2.95-10.09) | 5.91(3.37-10.16) |
| Fujian | 5.21(3.18-7.23) | 4.62(3.11-6.82) | 5.86(3.71-8) | 5.18(3.56-7.47) |
| Gansu | 8.75(2.56-14.94) | 8.32(3.99-16.54) | 6.25(2.06-13.99) | 5.84(2.44-13.37) |
| Guangdong | 5(3.91-6.08) | 4.27(3.42-5.32) | 5.39(4.26-6.51) | 4.57(3.69-5.65) |
| Guangxi | 6.21(3.64-8.79) | 5.39(3.52-8.16) | 4.73(2.47-7) | 4.05(2.49-6.54) |
| Guizhou | 4.94(1.6-8.27) | 4.56(2.29-8.88) | 4.94(1.6-8.27) | 4.5(2.26-8.78) |
| Hainan | 4.55(0.56-15.47) | 4.01(1-14.79) | 11.36(3.79-24.56) | 10.13(4.22-22.39) |
| Hebei | 12.79(11.66-13.93) | 11.92(10.87-13.05) | 12.67(11.54-13.8) | 11.8(10.75-12.93) |
| Heilongjiang | 4.02(2.89-5.16) | 4.05(3.04-5.37) | 5.34(4.03-6.64) | 5.35(4.18-6.82) |
| Henan | 3.48(2.97-4) | 3.33(2.87-3.87) | 4.08(3.52-4.63) | 3.89(3.39-4.46) |
| Hubei | 4.76(3.24-6.28) | 4.55(3.29-6.26) | 6.35(4.61-8.09) | 6.07(4.59-7.97) |
| Hunan | 3.2(1.55-4.84) | 2.76(1.64-4.62) | 5.71(3.54-7.88) | 4.92(3.33-7.21) |
| Jiangxi | 6.21(4.16-8.27) | 4.96(3.53-6.93) | 8.1(5.78-10.42) | 6.42(4.77-8.6) |
| Jiansu | 4.61(3.46-5.75) | 4.54(3.53-5.83) | 4.53(3.39-5.67) | 4.47(3.47-5.75) |
| Jilin | 3.8(2.21-5.4) | 4.03(2.64-6.1) | 4.53(2.79-6.26) | 4.83(3.28-7.05) |
| Liaoning | 4.8(3.2-6.39) | 4.97(3.55-6.92) | 3.92(2.47-5.38) | 4.09(2.82-5.9) |
| Neimenggu | 7.16(4.4-9.93) | 7.55(5.1-11.03) | 7.16(4.4-9.93) | 7.55(5.1-11.04) |
| Ningxia | 9.38(1.98-25.02) | 8.73(2.81-24.05) | 6.25(0.77-20.81) | 5.74(1.42-20.41) |
| Qinghai | - | - | - | - |
| Shananxi | 4.13(1.49-6.77) | 3.71(1.93-7) | 5.05(2.14-7.95) | 4.51(2.5-7.99) |
| Shandong | 2.93(2.01-3.85) | 3.02(2.2-4.12) | 3.24(2.28-4.2) | 3.34(2.47-4.49) |
| Shanghai | 5.93(4.05-7.81) | 5.85(4.24-8.02) | 6.43(4.47-8.38) | 6.34(4.66-8.58) |
| Shanxi | 7.55(6.33-8.77) | 7.51(6.37-8.84) | 7.77(6.53-9.02) | 7.76(6.6-9.1) |
| Sichuan | 4.88(2.68-7.08) | 4.53(2.86-7.09) | 5.15(2.89-7.4) | 4.75(3.04-7.35) |
| Tianjing | 18.7(17.54-19.86) | 16.92(15.83-18.06) | 17.48(16.35-18.61) | 15.76(14.71-16.87) |
| Xinjiang | - | - | 1.18(0.03-6.38) | - |
| Xizang | 12.5(1.55-38.35) | 9.82(2.4-32.59) | - | - |
| Yunnan | 3.01(0.41-5.61) | 2.9(1.21-6.79) | 2.41(0.08-4.74) | 2.29(0.86-5.97) |
| Zhejiang | 4.87(3.4-6.34) | 4.5(3.31-6.09) | 5.35(3.81-6.89) | 4.94(3.69-6.59) |

**Appendix Table 2 The sensitivity analyses of factors influence on COVID-19 Vaccine Hesitancy(n=24960).**

| **Covariates** | **Hesitancy rate in the first phase of vaccination** | | **Predicted hesitancy rate in the second phase of vaccination** | |
| --- | --- | --- | --- | --- |
|  | **Model 1** | **Model 2** | **Model 1** | **Model 2** |
| **Socio-demographic** |  |  |  |  |
| Age, years |  |  |  |  |
| 18-29 | 1.00(ref.) | 1.00(ref.) | 1.00(ref.) | 1.00(ref.) |
| 30-39 | 0.64(0.57-0.72)* | 0.81(0.68-0.95)* | 0.59(0.53-0.67)* | 0.78(0.66-0.91)* |
| 40-49 | 0.49(0.39-0.61)* | 0.82(0.63-1.08) | 0.37(0.29-0.47)* | 0.60(0.45-0.79)* |
| 50-59 | 0.42(0.28-0.64)* | 0.63(0.39-1.02) | 0.38(0.25-0.58)* | 0.58(0.36-0.93)* |
| 60- | 0.67(0.29-1.52) | 0.83(0.32-2.18) | 0.61(0.27-1.39) | 0.79(0.30-2.04) |
| Sex |  |  |  |  |
| Men | 1.00(ref.) | 1.00(ref.) | 1.00(ref.) | 1.00(ref.) |
| Women | 0.50(0.45-0.56)* | 0.78(0.68-0.90)* | 0.50(0.45-0.56)* | 0.75(0.66-0.85)* |
| Educational status |  |  |  |  |
| Some high school | 1.00(ref.) | 1.00(ref.) | 1.00(ref.) | 1.00(ref.) |
| High school graduate | 0.51(0.43-0.60)* | 0.72(0.59-0.88)* | 0.53(0.44-0.63)* | 0.71(0.58-0.87)* |
| University graduate | 0.39(0.34-0.46)* | 0.67(0.56-0.81)* | 0.49(0.42-0.57)* | 0.75(0.62-0.91)* |
| Ethnic groups |  |  |  |  |
| Han | 1.00(ref.) | 1.00(ref.) | 1.00(ref.) | 1.00(ref.) |
| Minority | 1.84(1.44-2.35)* | 1.19(0.89-1.60) | 1.38(1.06-1.80)* | 0.85(0.62-1.16) |
| Religion |  |  |  |  |
| Atheist | 1.00(ref.) | 1.00(ref.) | 1.00(ref.) | 1.00(ref.) |
| Others | 1.83(1.58-2.12)* | 1.13(0.95-1.35) | 1.41(1.21-1.65)* | 0.93(0.77-1.11) |
| Marital status |  |  |  |  |
| Married | 1.00(ref.) | 1.00(ref.) | 1.00(ref.) | 1.00(ref.) |
| Others | 1.74(1.56-1.94)* | 1.36(1.17-1.58)* | 1.87(1.68-2.08)* | 1.32(1.14-1.53)* |
| Score of health condition | 0.97(0.97-0.97)* | 1.00(0.99-1.00) | 0.97(0.97-0.97)* | 1.00(0.99-1.00)* |
| Subjective social status |  |  |  |  |
| In China | 1.05(1.03-1.08)* | 0.97(0.92-1.02) | 1.03(1.00-1.05) | 0.99(0.95-1.04) |
| In one’s Community | 1.07(1.04-1.10)* | 1.05(1.00-1.10)* | 1.03(1.01-1.06)* | 1.02(0.98-1.07) |
| Smoking status |  |  |  |  |
| Current smoker | 1.00(ref.) | 1.00(ref.) | 1.00(ref.) | 1.00(ref.) |
| Former smoker | 0.59(0.47-0.73)* | 1.12(0.86-1.47) | 0.46(0.36-0.59)* | 0.81(0.61-1.08) |
| Never smoker | 0.23(0.20-0.26)* | 0.64(0.54-0.75)* | 0.27(0.24-0.30)* | 0.72(0.61-0.84)* |
| Drinking status |  |  |  |  |
| Current drinker | 1.00(ref.) | 1.00(ref.) | 1.00(ref.) | 1.00(ref.) |
| Former drinker | 1.17(0.90-1.51) | 1.41(1.04-1.92)* | 0.92(0.69-1.21) | 1.03(0.75-1.42) |
| Never drinker | 0.41(0.36-0.47)* | 0.95(0.80-1.14) | 0.43(0.38-0.49)* | 0.89(0.76-1.05) |
| Health behaviors |  |  |  |  |
| Washing hands |  |  |  |  |
| Increased | 1.00(ref.) | 1.00(ref.) | 1.00(ref.) | 1.00(ref.) |
| Unchanged/Decreased | 4.57(4.09-5.11)* | 1.67(1.44-1.93)* | 4.31(3.87-4.81)* | 1.63(1.42-1.88)* |
| Wearing mask |  |  |  |  |
| Increased | 1.00(ref.) | 1.00(ref.) | 1.00(ref.) | 1.00(ref.) |
| Unchanged/Decreased | 7.83(6.86-8.93)* | 1.76(1.48-2.09)* | 6.70(5.87-7.65)* | 1.63(1.38-1.94)* |
| Social distance |  |  |  |  |
| Increased | 1.00(ref.) | 1.00(ref.) | 1.00(ref.) | 1.00(ref.) |
| Unchanged/Decreased | 5.07(4.36-5.88)* | 1.66(1.39-1.97)* | 4.04(3.53-4.63)* | 1.44(1.23-1.68)* |
| **Awareness of COVID-19 pandemic** |  |  |  |  |
| COV**ID-19 conspiracy beliefs** |  |  |  |  |
| Level 1 | 1.00(ref.) | 1.00(ref.) | 1.00(ref.) | 1.00(ref.) |
| Level 2 | 1.15(0.92-1.43) | 0.99(0.77-1.27) | 1.14(0.92-1.40) | 0.91(0.72-1.15) |
| Level 3 | 2.21(1.83-2.67)* | 1.07(0.83-1.37) | 2.37(1.98-2.83)* | 1.03(0.82-1.31) |
| Level 4 | 4.79(4.03-5.70)* | 1.17(0.90-1.53) | 4.33(3.66-5.12)* | 1.00(0.77-1.28) |
| Risk of COVID-19 infection |  |  |  |  |
| Very high | 1.00(ref.) | 1.00(ref.) | 1.00(ref.) | 1.00(ref.) |
| High | 2.03(1.61-2.55)* | 1.56(1.20-2.02)* | 2.17(1.70-2.76)* | 1.64(1.25-2.14)* |
| Medium | 1.20(1.00-1.49) | 1.30(1.01-1.67)* | 1.40(1.12-1.76)* | 1.44(1.12-1.86)* |
| Low | 0.39(0.32-0.48)* | 0.81(0.64-1.04) | 0.53(0.43-0.65)* | 1.01(0.79-1.30) |
| No | 0.49(0.38-0.62)* | 0.88(0.67-1.17) | 0.55(0.43-0.71)* | 0.94(0.71-1.25) |
| Not sure | 1.19(0.89-1.61) | 0.82(0.54-1.24) | 1.60(1.19-2.15)* | 1.05(0.70-1.56) |
| Curability of COVID-19 |  |  |  |  |
| Very high | 1.00(ref.) | 1.00(ref.) | 1.00(ref.) | 1.00(ref.) |
| High | 1.90(1.63-2.21)* | 1.32(1.12-1.56)* | 1.82(1.57-2.11)* | 1.22(1.04-1.43)* |
| Medium | 6.33(5.40-7.42)* | 2.61(2.17-3.14)* | 5.78(4.94-6.77)* | 2.32(1.94-2.78)* |
| Low | 5.59(4.56-6.87)* | 2.74(2.16-3.48)* | 6.63(5.47-8.03)* | 3.35(2.69-4.18)* |
| No | 3.87(2.72-5.51)* | 1.76(1.15-2.70)* | 4.70(3.41-6.49)* | 2.64(1.81-3.85)* |
| Not sure | 4.13(3.11-5.48)* | 2.25(1.55-3.28)* | 4.00(3.02-5.28)* | 1.96(1.36-2.83)* |
| **COVID-19 vaccine exception** |  |  |  |  |
| Channel of vaccine information |  |  |  |  |
| Community worker | 1.00(ref.) | 1.00(ref.) | 1.00(ref.) | 1.00(ref.) |
| Internet | 1.54(1.32-1.79)* | 1.39(1.17-1.65)* | 1.56(1.34-1.81)* | 1.33(1.13-1.57)* |
| Others | 3.82(3.26-4.49)* | 2.57(2.14-3.08)* | 3.51(3.00-4.11)* | 2.24(1.88-2.68)* |
| Vaccine conspiracy beliefs |  |  |  |  |
| Level 1 | 1.00(ref.) | 1.00(ref.) | 1.00(ref.) | 1.00(ref.) |
| Level 2 | 0.97(0.77-1.22) | 1.04(0.80-1.35) | 1.04(0.83-1.30) | 1.19(0.92-1.53) |
| Level 3 | 1.85(1.52-2.25)* | 1.09(0.85-1.40) | 2.01(1.66-2.44)* | 1.34(1.06-1.71)* |
| Level 4 | 6.60(5.53-7.87)* | 1.38(1.05-1.81)* | 6.76(5.67-8.05)* | 1.85(1.42-2.40)* |
| Weigh risks of vaccination against risks of the disease |  |  |  |  |
| Disease outweigh vaccine | 1.00(ref.) | 1.00(ref.) | 1.00(ref.) | 1.00(ref.) |
| Vaccine outweigh disease | 2.82(2.52-3.15)* | 1.32(1.16-1.50)* | 2.91(2.61-3.25)* | 1.41(1.24-1.59)* |
| Other life/health responsibilities |  |  |  |  |
| Very high | 1.00(ref.) | 1.00(ref.) | 1.00(ref.) | 1.00(ref.) |
| High | 1.61(1.35-1.92)* | 0.91(0.75-1.11) | 2.21(1.88-2.61)* | 1.25(1.04-1.50)* |
| Medium | 10.40(8.90-12.17)* | 2.37(1.94-2.88)* | 11.48(9.84-13.40)* | 2.67(2.19-3.24)* |
| Low | 15.95(13.22-19.25)* | 3.69(2.97-4.59)* | 12.45(10.24-15.14)* | 2.92(2.33-3.65)* |
| Very low | 10.38(8.22-13.10)* | 4.04(3.11-5.25)* | 8.63(6.77-11.01)* | 3.40(2.60-4.46)* |
| Convenient vaccination |  |  |  |  |
| High | 1.00(ref.) | 1.00(ref.) | 1.00(ref.) | 1.00(ref.) |
| Medium | 3.59(2.99-4.32)* | 1.45(1.16-1.82)* | 3.39(2.82-4.07)* | 1.34(1.07-1.66)* |
| Low | 5.06(3.68-6.97)* | 2.29(1.53-3.42)* | 4.90(3.57-6.73)* | 2.14(1.45-3.17)* |
| **Trust in healthcare system** |  |  |  |  |
| Trust in doctors |  |  |  |  |
| Level 1 | 1.00(ref.) | 1.00(ref.) | 1.00(ref.) | 1.00(ref.) |
| Level 2 | 0.33(0.29-0.38)* | 0.73(0.61-0.87)* | 0.36(0.31-0.41)* | 0.80(0.67-0.94)* |
| Level 3 | 0.18(0.15-0.22)* | 0.68(0.54-0.86)* | 0.17(0.14-0.20)* | 0.63(0.50-0.79)* |
| Level 4 | 0.08(0.06-0.10)* | 0.70(0.51-0.97)* | 0.08(0.06-0.10)* | 0.72(0.53-0.98)* |
| Trust in developers |  |  |  |  |
| Level 1 | 1.00(ref.) | 1.00(ref.) | 1.00(ref.) | 1.00(ref.) |
| Level 2 | 0.27(0.24-0.31)* | 0.61(0.51-0.72)* | 0.26(0.23-0.29)* | 0.57(0.49-0.67)* |
| Level 3 | 0.07(0.06-0.09)* | 0.51(0.38-0.67)* | 0.08(0.07-0.10)* | 0.53(0.41-0.69)* |

* *P*<0.05

We categorized the score of COVID-19 conspiracy beliefs by quartiles as level 1 (≤7 points), level 2 (8-13 points), level 3 (14-20 points), level 4 (≥21 points); the score of vaccine conspiracy beliefs by quartiles as level 1 (≤7 points), level 2 (8-12 points), level 3 (13-18 points), level 4 (≥19 points); We categorized the score of trust in doctors by quartiles as level 1 (≤30 points), level 2 (31-34 points), level 3 (35-38 points), level 4 (≥39 points); the score of trust in developers by quartiles as level 1 (≤17 points), level 2 (18-21 points), level 3 (≥22 points).

Model 1: unadjusted;

Model 2: adjusted age, sex, educational status, ethnic groups, religion, marital status, change one’s job, family doctor, score of health condition, subjective social status in China, subjective social status in one’s community, body mass index, chronic condition, smoking status, drinking status, health behaviors, COVID-19 conspiracy beliefs, risk of COVID-19 infection, curability of COVID-19, channel of vaccine information, vaccine conspiracy beliefs, weigh risks of vaccination against risks of the disease, other life/health responsibilities, trust in doctors, trust in developers, convenient vaccination.
